# Supplementary material for: Targeted protein degradation via intramolecular bivalent glues
Source: Nature. 2024 Feb 21;627(8002):204–11. doi: 10.1038/s41586-024-07089-6 (PMC10917667; doi:10.1038/s41586-024-07089-6)
Supplement: Supplementary file 1 — Supplementary Methods details chemical synthesis of compounds; Supplementary Fig. 1 displays uncropped western blot and SDS–PAGE images. Supplementary Fig. 2 shows gating strategies for flow cytometric analyses and cell sorting. [file 41586_2024_7089_MOESM1_ESM.pdf]

nature portfolio

https://doi.org/10.1038/s41586-024-07089-6

Supplementary information

Targeted protein degradation via intramolecular bivalent glues

In the format provided by the authors and unedited

Supplementary Information for

Targeted protein degradation via intramolecular bivalent glues

This file contains:

Supplementary Methods

Supplementary Figure 1 | Uncropped western blots and SDS-PAGE images.

Supplementary Figure 2 | Gating strategies for flow cytometric analyses and cell sorting.

Supplementary Methods

Chemical Synthesis

Chemicals that are commercially available were purchased from Apollo Scientific, Sigma-Aldrich, Fluorochem, CombiBlocks, TCI, and Enamine and were used without further purification. Liquid chromatography–mass spectrometry (LC-MS) was carried out on a Shimadzu HPLC/MS 2020 equipped with a Hypersil Gold column (1.9 μm, 50 × 2.1 mm^2), a photodiode array detector, and an electrospray ionization (ESI) detector. The samples were eluted with a 3 min gradient of 5–95% acetonitrile in water containing 0.1% formic acid at a flow rate of 0.8 mL/min. Flash column chromatography was performed on a Teledyne ISCO Combiflash Companion installed with disposable normal phase RediSep Rf columns (230–400 mesh, 40–63 mm; SiliCycle). Preparative HPLC purification was performed on a Gilson preparative HPLC system equipped with a Waters X-Select C18 column (100 mm × 19 mm and 5 μm particle size) using a gradient from 5 to 95% of acetonitrile in water containing 0.1% formic acid over 10 min at a flow rate of 25 mL/min. Compound characterization using NMR was performed either on a Bruker 500 Ultra shield or on a Bruker Ascend 400 spectrometer. The ^{1}H NMR and ^{13}C NMR reference solvents used are CDCl3-*d1* (δH = 7.26 ppm/δC = 77.16 ppm), CD3OD (δH = 3.34 ppm/δC = 49.86 ppm), DMSO-*d6* (δH = 2.50 ppm/δC = 39.52 ppm), or acetone-*d6* (δH = 2.05 ppm/δC = 29.84 ppm). Signal patterns are described as singlet (s), doublet (d), triplet (t), quartet (q), multiplet (m), broad singlet (bs), or a combination of the listed splitting patterns. The coupling constants (*J*) are measured in hertz (Hz).

Synthesis of IBG1

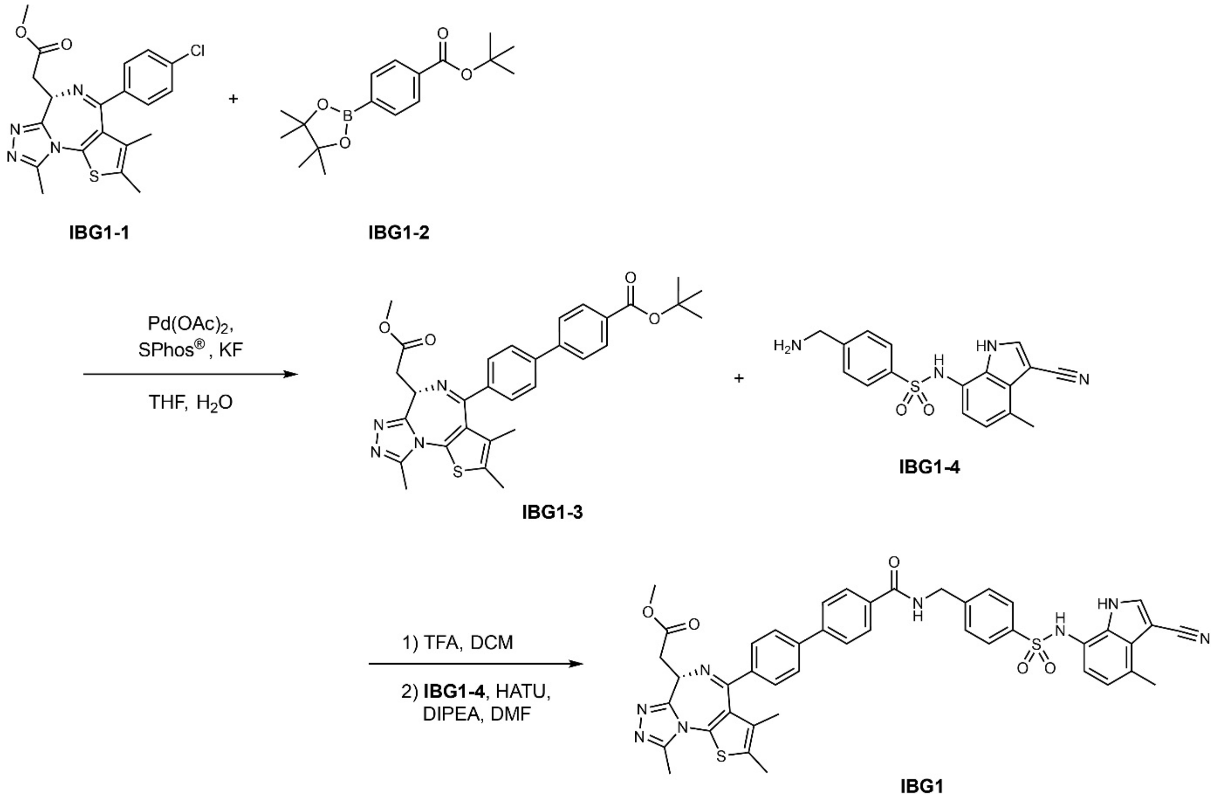

tert-Butyl (S)-4'-(6-(2-methoxy-2-oxoethyl)-2,3,9-trimethyl-6H-thieno[3,2-f][1,2,4]triazolo[4,3-a][1,4]diazepin-4-yl)-[1,1'-biphenyl]-4-carboxylate (**IBG1-3**)

A mixture of methyl (R)-2-(4-(4-chlorophenyl)-2,3,9-trimethyl-6H-thieno[3,2-f][1,2,4]triazolo[4,3-a]azepin-6-yl)acetate (**IBG1-1**) (126 mg, 0.30 mmol), 4-(*tert*-utoxycarbonyl)phenylboronic acid pinacol ester (**IBG1-2**) (101 mg, 0.33 mmol, 1.1 eq.), potassium fluoride (52.8 mg, 0.91 mmol, 3.0 eq.), SPhos® (12.4 mg, 0.03 mmol, 0.1 eq.), palladium(II) acetate (6.8 mg, 0.03 mmol, 0.1 eq.), and water (20 µL) in THF (1.0 mL) was purged with nitrogen atmosphere then the mixture was stirred at reflux temperature overnight. The resulted mixture was diluted with excess amount of EtOAc, filtered through a celite pad, washed with EtOAc, concentrated in vacuo, and then purified by silica gel column chromatography (DCM-MeOH) to afford **IBG1-3** (193 mg, quantitative yield.). ^{1}H NMR (500 MHz, $CDCl_3$) δ 8.05 (d, *J* = 8.4 Hz, 2H), 7.63-7.59 (m, 4H), 7.56-7.54 (m, 2H), 4.66 (dd, *J* = 7.8, 6.3 Hz, 1H), 3.79 (s, 3H), 3.72-3.62 (m, 2H), 2.69 (s, 3H), 2.43 (s, 3H), 1.75 (s, 3H), 1.61 (s, 9H). ^{13}C NMR (101 MHz, CD_3OD) δ 174.08, 167.99, 167.91, 157.75, 153.06, 146.32, 144.46, 139.95, 134.31, 134.10, 133.41, 133.22, 133.14, 131.89, 131.33, 129.23, 128.93, 83.33, 55.82, 53.31, 38.11, 29.34, 15.29, 13.83, 12.48. LC-MS, ESI$^+$, *m/z* 557.4 $[M+H]^+$.

Methyl (S)-2-(4-(4'-((4-(*N*-(3-cyano-4-methyl-1H-indol-7-yl)sulfamoyl)benzyl)carbamoyl)-[1,1'-biphenyl]-4-yl)-2,3,9-trimethyl-6H-thieno[3,2-f][1,2,4]triazolo[4,3-a][1,4]diazepin-6-yl)acetate (**IBG1**)

To a solution of **IBG1-3** (57.0 mg, 90 µmol) in DCM (0.5 mL) was added trifluoroacetic acid (0.5 mL) and then the mixture was stirred at room temperature for 4 hours. The resulted mixture was concentrated in vacuo, and then toluene was added thereto. After concentrated in vacuo again, the obtained crude compound, 4-(aminomethyl)-*N*-(3-cyano-4-methyl-1H-indol-7-yl)benzenesulfonamide (**IBG1-4**)[6] (30.8 mg, 90.5 µmol, 1.0 eq.), and *N,N*-diisopropylethylamine (78.8 µL, 453 µmol, 5.0 eq.) were mixed in *N*,*N*-dimethylformamide (1.0 mL) and then HATU (61.9 mg, 163 µmol, 1.8 eq.) was added to it. After the mixture was stirred at room temperature overnight, the resulted mixture was purified with preparative HPLC (ODS, H_2O-MeCN with 0.1% HCOOH) to afford **IBG1** (26.2 mg, 35% yield). ^{1}H NMR (500 MHz, DMSO-*d6*) δ 11.86 (d, *J* = 2.7 Hz, 1H), 9.90 (s, 1H), 9.14 (t, J = 6.0 Hz, 1H), 8.15 (d, *J* = 3.1 Hz, 1H), 8.00 (d, J = 8.4 Hz, 2H), 7.83-7.79 (m, 4H), 7.69 (d, *J* = 8.4 Hz, 2H), 7.53 (d, *J* = 8.3 Hz, 2H), 7.45 (d, *J* = 8.3 Hz, 2H), 6.77 (d, *J* = 7.8 Hz, 1H), 6.62 (d, 7.8 Hz, 1H), 4.55-4.52 (m, 3H), 3.69 (s, 3H), 3.54-3.43 (m, 2H), 2.62 (s, 3H), 2.56 (s, 3H), 2.43 (s, 3H), 1.69 (s, 3H). ^{13}C NMR (126 MHz, DMSO-*d6*) δ 171.07, 165.87, 163.87, 154.72, 149.89, 144.91, 141.80, 140.90, 137.67, 137.33, 135.14, 133.26, 132.03, 130.71, 130.36, 130.04, 129.84, 129.00, 127.99, 127.54, 127.26, 127.02, 126.76, 126.63, 126.44, 122.40, 120.55, 118.01, 117.29, 84.30, 53.42, 51.51, 42.24, 36.28, 17.56, 14.04, 12.65, 11.21. HRMS, ESI$^+$, *m/z* calcd for C44H39N8O5S2 [M+H]$^+$ 823.2485; found 823.2505.

Synthesis of compound 1a

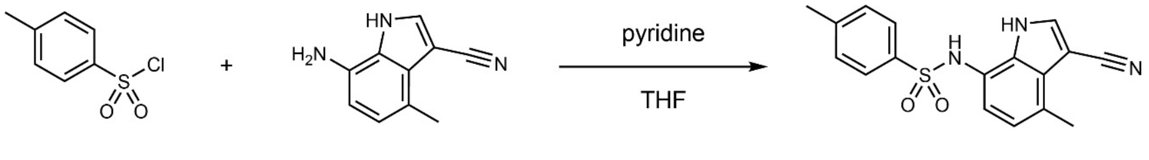

1a-1 **1a**

<u>*N*-(3-Cyano-4-methyl-1H-indol-7-yl)-4-methylbenzenesulfonamide (**1a**)</u>

To a solution of 7-amino-4-methyl-1H-indole-3-carbonitrile (**1a-1**) (30 mg, 0.18 mmol) and pyridine (42.5 µL, 0.53 mmol, 3.0 eq.) in tetrahydrofuran (1.0 mL) was added 4-toluenesulfonyl chloride (50.1 mg, 0.26 mmol, 1.5 eq.) and then the mixture was stirred at room temperature overnight. The resulted mixture was diluted with DMSO (1.0 mL), filtered through a membrane filter (0.43 µm), and then purified by preparative HPLC (ODS, H_2O-MeCN with 0.1% HCOOH) to afford **1a** (39.3 mg, 0.117 mmol in 71% yield). ^{1}H NMR (400 MHz, DMSO-*d6*) δ 11.85 (bs, 1H), 9.82 (bs, 1H), 8.15 (s, 1H), 7.58 (d, *J* = 8.3 Hz, 2H), 7.31 (d, *J* = 8.1 Hz, 2H), 6.77 (d, J = 7.7 Hz, 1H), 6.60 (d, *J* = 7.7 Hz, 1H), 2.56 (s, 3H), 2.34 (s, 3H). ^{13}C NMR (101 MHz, DMSO-*d4*) δ 144.87, 138.10, 136.81, 132.13, 131.18, 128.89, 128.59, 128.11, 124.09, 122.37, 119.79, 119.01, 85.98, 22.60, 19.26. LC-MS, ESI$^+$, *m/z* 323.8 [M+H]$^+$.

Synthesis of compound 1b

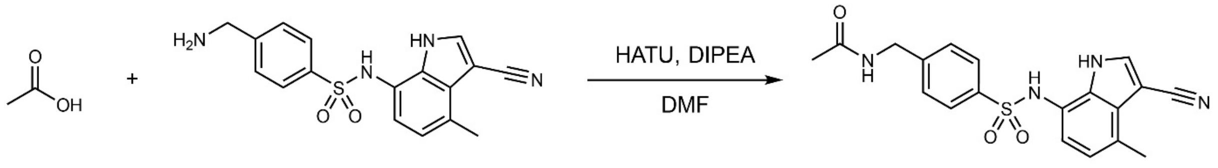

IBG1-4 **1b**

N-(4-(*N*-(3-Cyano-4-methyl-1H-indol-7-yl)sulfamoyl)benzyl)acetamide (**1b**)

To a solution of 4-(aminomethyl)-*N*-(3-cyano-4-methyl-1H-indol-7-yl)benzenesulfonamide (**IBG1-4**) (20 mg, 58.8 µmol, 1.0 eq.), acetic acid (5.0 µL, 456 µmol, 5.0 eq.), and *N,N*-diisopropylethylamine (51.2 µL, 294 µmol, 5.0 eq.) in *N,N*-dimethylformamide (0.5 mL) was added HATU (40.2 mg, 106 µmol, 1.8 eq.) and then the mixture was stirred at room temperature for 6 hours. The resulted mixture was diluted with 0.5 mL of DMSO, filtered, and purified with preparative HPLC (ODS, H_2O-MeCN with 0.1% HCOOH) to afford **1b** (3.4 mg, 15% yield). ^{1}H NMR (400 MHz, acetone-*d6*) δ 8.05 (s, 1H), 7.62 (d, *J* = 8.3 Hz, 2H), 7.39 (d, *J* = 8.3 Hz, 2H), 6.78 (d, *J* = 7.7 Hz, 1H), 6.63 (d, *J* = 7.7 Hz, 1H), 4.41 (s, 2H), 2.64 (s, 3H), 1.95 (s, 3H). ^{13}C NMR (101 MHz, acetone-*d6*) δ 171.07, 146.14, 138.80, 135.07, 132.41, 129.81, 128.64, 128.35, 127.96, 123.61, 121.37, 120.96, 100.21, 86.66, 43.08, 22.71, 18.20. LC-MS, ESI$^+$, *m/z* 382.9 [M+H]$^+$.

Synthesis of compound 1c

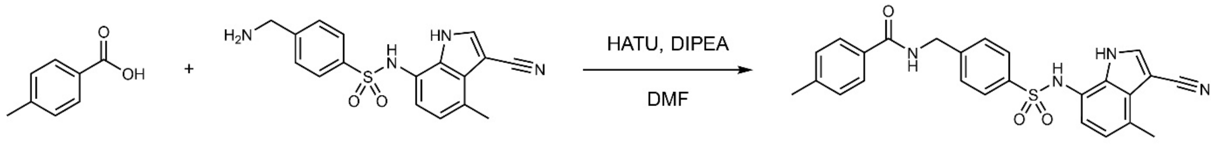

IBG1-4 **1c**

N-(4-(*N*-(3-Cyano-4-methyl-1H-indol-7-yl)sulfamoyl)benzyl)-4-methylbenzamide (**1c**)

To a solution of 4-(aminomethyl)-*N*-(3-cyano-4-methyl-1H-indol-7-yl)benzenesulfonamide (**IBG1-4**) (30.0 mg, 79.6 µmol), *p*-toluic acid (13.0 mg, 95.5 µmol, 5.0 eq.), and *N,N*-diisopropylethylamine (69.3 µL, 398 µmol, 5.0 eq.) in *N,N*-dimethylformamide (0.5 mL) was added HATU (45.4 mg, 119 µmol, 1.5 eq.) and then the mixture was stirred at room temperature for 5 hours. The resulted mixture was purified with silica gel column chromatography (ODS, H_2O-MeCN with 0.1% HCOOH). The obtained compound was triturated with DCM. The solid was collected by filtration, washed with DCM, and then dried in vacuo to afford **1c** (4.8 mg, 13% yield). ^{1}H NMR (500 MHz, acetone-*d6*) δ 11.09 (bs, 1H), 8.87 (bs, 1H), 8.28 (bs, 1H), 8.09 (s, 1H), 7.83 (d, *J* = 8.2 Hz, 2H), 7.65 (d, *J* = 8.4 Hz, 2H), 7.48 (d, *J* = 8.4 Hz, 2H), 7.28 (d, *J* = 8.0 Hz, 2H), 6.80 (dd, *J* = 7.1, 0.7 Hz, 1H), 6.67 (d, *J* = 7.7 Hz, 1H), 4.63 (d, *J* = 6.0

Hz, 2H), 2.64 (s, 3H), 2.37 (s, 3H). ^{13}C NMR (126 MHz, acetone-*d6*) δ 167.36, 146.43, 142.49, 138.64, 135.15, 132.71, 132.44, 129.83, 129.75, 128.67, 128.30, 128.13, 127.90, 123.57, 121.37, 120.96, 117.56, 86.73, 43.43, 21.34, 18.21. LC-MS, ESI$^+$, *m/z* 459.1 [M+H]$^+$.

Synthesis of compound 1d

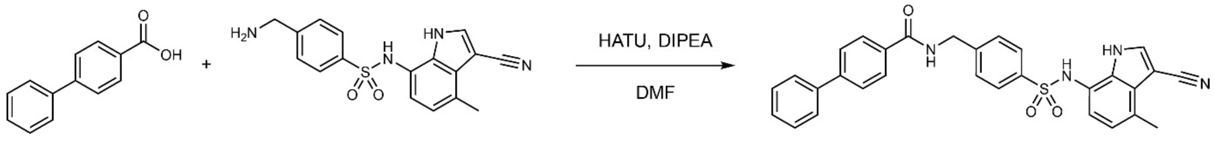

N-(4-(*N*-(3-Cyano-4-methyl-1H-indol-7-yl)sulfamoyl)benzyl)-[1,1'-biphenyl]-4-carboxamide (**1d**)

To a solution of 4-(aminomethyl)-*N*-(3-cyano-4-methyl-1H-indol-7-yl)benzenesulfonamide hydrochloride (**IBG1-4**) (30.0 mg, 79.6 μmol), 4-phenylbenzoic acid (18.9 mg, 95.5 μmol, 5.0 eq.), and *N,N*-diisopropylethylamine (69.3 μL, 398 μmol, 5.0 eq.) in *N,N*-dimethylformamide (0.5 mL) was added HATU (45.4 mg, 119 μmol, 1.5 eq.) and then the mixture was stirred at room temperature for 5 hours. The resulted mixture was purified with silica gel column chromatography (ODS, H_2O-MeCN with 0.1% HCOOH). The obtained compound was triturated with DCM. The solid was collected by filtration, washed with DCM, and then dried in vacuo to afford **1d** (3.69 mg, 9% yield). ^{1}H NMR (500 MHz, DMSO-*d6*) δ 11.91 (s, 1H), 9.94 (s, 1H), 9.16 (t, *J* = 6.0 Hz, 1H), 8.16 (s, 1H), 7.99 (d, *J* = 8.5 Hz, 2H), 7.79 (d, *J* = 8.4 Hz, 2H), 7.75-7.73 (m, 2H), 7.68 (d, *J* = 8.4 Hz, 2H), 7.51-7.48 (m, 2H), 7.45 (d, *J* = 8.3 Hz, 2H), 7.41 (t, *J* = 7.4 Hz, 1H), 6.77 (d, *J* = 7.8 Hz, 1H), 6.59 (d, *J* = 7.8 Hz, 1H), 4.55 (d, *J* = 4.6 Hz, 2H), 2.55 (s, 3H). ^{13}C NMR (126 MHz, DMSO-*d6*) δ 165.98, 144.96, 142.93, 139.13, 135.21, 132.79, 130.49, 129.03, 128.07, 127.96, 127.52, 127.06, 126.87, 126.58, 126.47, 122.43, 118.11, 117.38, 84.28, 42.23, 17.64. LC-MS, ESI$^+$, *m/z* 521.1 [M+H]$^+$.

Synthesis of compound 1e

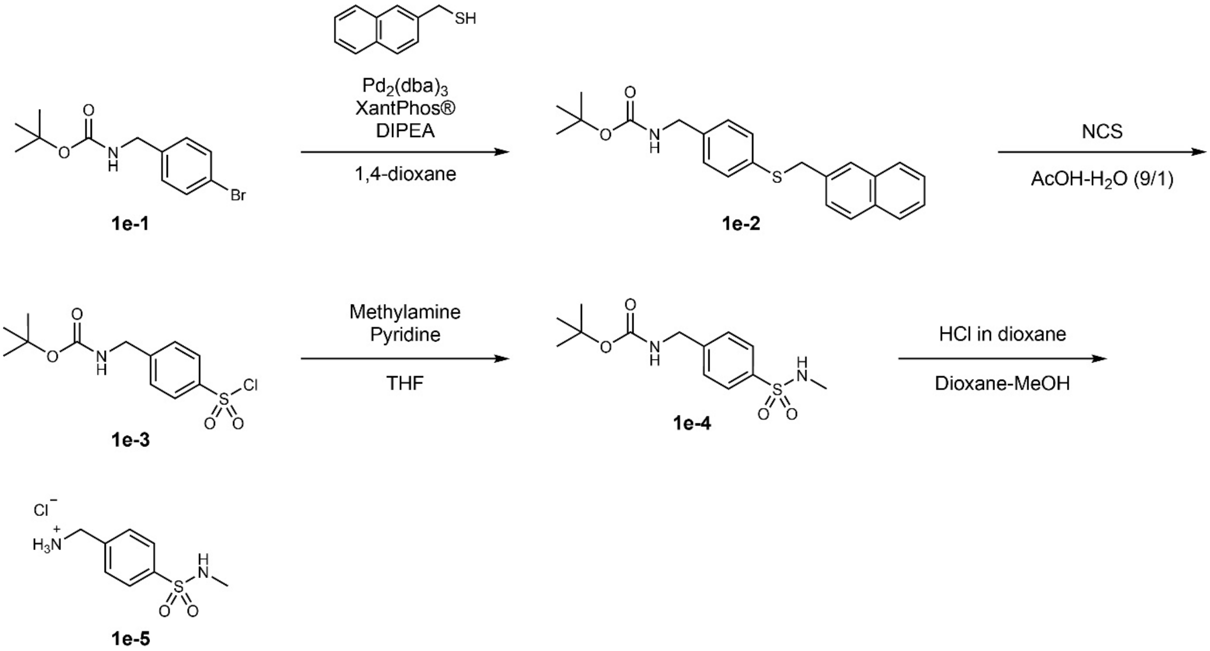

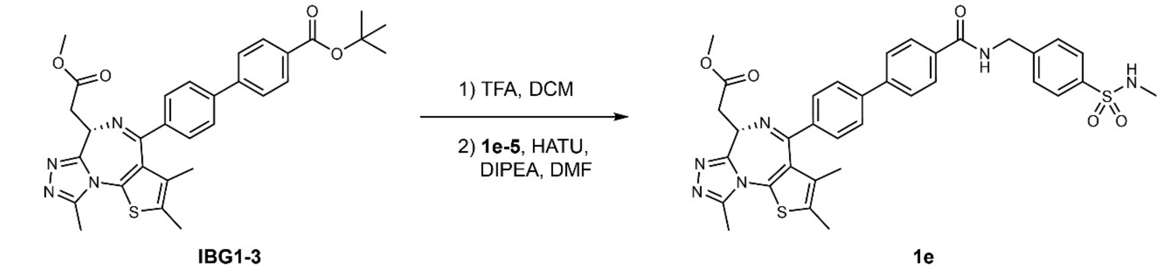

<u>*tert*-Butyl (4-((naphthalen-2-ylmethyl)thio)benzyl)carbamate (**1e-2**)</u>

To a mixture of *tert*-butyl bromobenzylcarbamate (**1e-1**) (500 mg, 1.75 mmol), naphthalen-2-ylmethanethiol (335 mg, 1.92 mmol, 1.1 eq.), tris(dibenzylideneacetone)dipalladium(0) (80.0 mg, 0.09 mmol, 0.05 eq.), XantPhos® (101 mg, 0.17 mmol, 0.1 eq.) was added DIPEA (609 µL, 3.49 mmol, 2.0 eq.) and 1,4-dioxane (10 mL) under nitrogen and then the mixture was stirred at 100 °C for 8 hours. The resulted mixture was concentrated in vacuo and then purified by silica gel column chromatography (heptane-EtOAc) to afford **1e-2** (584 mg, 88% yield). ^{1}H NMR (400 MHz, $CDCl_3$) δ 7.81-7.73 (m, 3H), 7.67 (s, 1H), 7.47-7.44 (m, 3H), 7.28 (d, *J* = 8.2 Hz, 2H), 7.15 (d, *J* = 7.9 Hz, 2H), 4.76 (bs, 1H), 4.25 (s, 4H), 1.45 (s, 9H). ^{13}C NMR (101 MHz, $CDCl_3$) δ 155.96, 137.56, 135.24, 135.04, 133.46, 132.75, 130.55, 128.44, 128.11, 127.83, 127.79, 127.53, 127.08, 126.28, 125.96, 79.70, 44.40, 39.72, 28.54. LC-MS, ESI$^-$, *m/z* 377.9 [M-H]$^-$.

<u>*tert*-Butyl (4-(chlorosulfonyl)benzyl)carbamate (**1e-3**)</u>

To a solution of **1e-2** (250 mg, 659 μmol) in acetic acid (9.0 mL) and water (1.0 mL) was added *N*-chlorosuccinimide (440 mg, 3.29 mmol, 5.0 eq.) and then the mixture was stirred at room temperature for 1.5 hours. The resulted mixture was added to water and then the organic was extracted with toluene. The organic layer was washed with water and brine, dried over $MgSO_4$, and then purified by silica gel column chromatography (heptane-EtOAc) to afford **1e-3** (57.6 mg, 29% yield). ^{1}H NMR (400 MHz, $CDCl_3$) δ 7.98 (d, *J* = 8.3 Hz, 2H), 7.52 (d, *J* = 8.3 Hz, 2H), 5.08 (bs, 1H), 4.42-4.41 (m, 2H), 1.46 (s, 9H). ^{13}C NMR (101 MHz, $CDCl_3$) δ 155.99, 147.73, 143.36, 128.30, 127.53, 80.43, 44.25, 28.50. LCMS was only detected as sulfonic acid form: LC-MS, ESI$^-$, *m/z* 285.8 $[M-Cl +O]^-$.

tert-Butyl (4-(*N*-methylsulfamoyl)benzyl)carbamate (**1e-4**)

To a solution of **1e-3** (22.1 mg, 72.3 μmol) and pyridine (17.5 μL, 217 μmol, 3.0 eq.) in tetrahydrofuran (1.0 mL) was added 2.0 M methylamine in THF (72.3 μL, 145 μmol, 2.0 eq.) and then the mixture was stirred at room temperature overnight. After the addition of 2.0 M methylamine in THF (72.3 μL, 145 μmol, 2.0 eq.), the mixture was stirred for 2 hours. The resulted mixture was added to water and then the organic was extracted with toluene. The organic layer was washed with water and brine, dried over $MgSO_4$, and then purified by silica gel column chromatography (heptane-EtOAc) to afford **1e-4** (17.6 mg, 81% yield). ^{1}H NMR (400 MHz, CD_3OD) δ 7.78 (d, *J* = 6.4 Hz, 2H), 7.46 (d, *J* = 6.4 Hz, 2H), 4.29 (s, 2H), 2.5 (s, 3H), 1.42 (s, 9H). ^{13}C NMR (101 MHz, $CDCl_3$) δ 155.83, 147.62, 143.12, 128.12, 127.31, 80.23, 44.04, 28.31. LC-MS, ESI$^+$, *m/z* 299.6 $[M+H]^+$.

4-(Aminomethyl)-*N*-methylbenzenesulfonamide hydrochloride (**1e-5**)

To a solution of **1e-4** (17.6 mg, 58.6 μmol) in 1,4-dioxane (293 μL) was added 4 M hydrogen chloride in 1,4-dioxane (293 μL, 1.17 mmol, 20 eq.), and then the mixture was stirred at room temperature for 3.5 hours. Additional 4 M hydrogen chloride in 1,4-dioxane (293 μL, 1.17 mmol, 20 eq.) and MeOH (293 μL) were added, and the mixture was stirred for 2 hours. The resulted mixture was concentrated in vacuo and then used for next reaction without further purification.

Methyl (S)-2-(4-(4'-(ethylcarbamoyl)-[1,1'-biphenyl]-4-yl)-2,3,9-trimethyl-6H-thieno[3,2-f][1,2,4]triazolo[4,3-a][1,4]diazepin-6-yl)acetate (**1e**)

To a solution of **IBG1-3** (18.2 mg, 36.4 μmol) in DCM (0.5 mL) was added trifluoroacetic acid (0.5 mL) and then the mixture was stirred at room temperature for 2 hours. The resulted mixture was concentrated in vacuo, and then toluene was added thereto. After concentrated in vacuo again to afford the corresponding carboxylic acid. To a mixture of the carboxylic acid, 4-(aminomethyl)-*N*-methylbenzenesulfonamide hydrochloride (**1e-5**) (13.8 mg, 58.2 μmol, 1.6 eq.), and *N,N*-diisopropylethylamine (31.7 μL, 182 μmol, 5.0 eq.) in *N,N*-dimethylformamide (1.0 mL) was added HATU (24.9 mg, 65.4 μmol, 1.5 eq.) and then the mixture was stirred at room temperature overnight. After the addition of HATU (24.9 mg, 65.4 μmol, 1.5 eq.) and *N,N*-diisopropylethylamine (31.7 μL, 182 μmol, 5.0 eq.), the mixture was stirred for 2 hours. The resulted mixture was added to ammonium chloride aqueous solution and then the

organic was extracted with EtOAc. The obtained organic extract was washed with brine, dried over $MgSO_4$, concentrated in vacuo, and then purified by silica gel column chromatography (DCM-MeOH). The obtained compound was purified again by preparative HPLC (ODS, H_2O-MeCN with 0.1% HCOOH) to afford **1e** (6.8 mg, 27% yield). ^{1}H NMR (400 MHz, CD_3OD) δ 8.00-7.98 (m, 2H), 7.84-7.79 (m, 4H), 7.77-7.75 (m, 2H), 7.59-7.58 (m, 4H), 4.70 (s, 2H), 4.66 (t, J = 7.2 Hz, 1H), 4.52 (s, 1H), 3.79 (s, 3H), 3.58 (d, *J* = 7.2 Hz, 2H), 2.73 (s, 3H), 2.53 (s, 3H), 2.49 (s, 3H), 1.76 (s, 3H). ^{13}C NMR (101 MHz, CD_3OD) δ 171.78, 168.40, 165.71, 155.44, 150.81, 144.09, 143.18, 142.16, 137.96, 137.56, 133.22, 132.02, 131.84, 130.90, 130.84, 129.07, 127.72, 127.68, 127.07, 126.89, 126.84, 53.48, 51.03, 42.71, 35.75, 27.80, 13.01, 11.53, 10.19. HRMS, ESI^{+}, *m/z* calcd for C35H35N6O5S2 $[M+H]^+$, 683.2110; found 683.215.

Synthesis of compound 1f

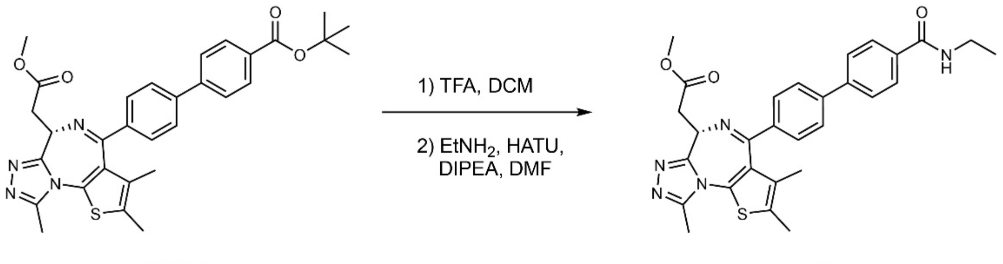

IBG1-3 **1f**

<u>Methyl (S)-2-(4-(4'-(ethylcarbamoyl)-[1,1'-biphenyl]-4-yl)-2,3,9-trimethyl-6H-thieno[3,2-f][1,2,4]triazolo[4,3-a][1,4]diazepin-6-yl)acetate (**1f**)</u>

To a solution of *tert*-butyl (S)-4'-(6-(2-methoxy-2-oxoethyl)-2,3,9-trimethyl-6H-thieno[3,2-f][1,2,4] triazolo[4,3-a][1,4]diazepin-4-yl)-[1,1'-biphenyl]-4-carboxylate (**IBG1-3**) (20.0 mg, 0.03 mmol) in DCM (0.5 mL) was added trifluoroacetic acid (0.5 mL) and then the mixture was stirred at room temperature for 2 hours. The resulted mixture was concentrated in vacuo, and then toluene was added thereto. After concentrated in vacuo again to afford the corresponding carboxylic acid. To the mixture of the carboxylic acid, 2 M ethylamine in THF (49.9 µL, 100 µmol, 2.0 eq.), and *N,N*-diisopropylethylamine (43.5 µL, 250 µmol, 5.0 eq.) in *N,N*-dimethylformamide (1.0 mL) was added HATU (28.5 mg, 74.9 µmol, 1.5 eq.) and then the mixture was stirred at room temperature overnight. The resulted mixture was purified directly by preparative HPLC (ODS, H_2O-MeCN with 0.1% HCOOH) to afford **1f** (4.9 mg, 19% yield) as a white solid. ^{1}H NMR (400 MHz, CD_3OD) δ 7.91 (d, *J* = 8.4, 2H), 7.77-7.72 (m, 4H), 7.56 (d, *J* = 8.2 Hz, 2H), 4.64 (t, *J* = 7.2 Hz, 1H), 4.50 (s, 1H), 3.78 (s, 3H), 3.56 (d, *J* = 7.2 Hz, 2H), 3.43 (q, *J* = 7.2 Hz, 2H), 2.71 (s, 3H), 2.47 (s, 3H), 1.75 (s, 3H), 1.24 (t, *J* = 7.2 Hz, 3H). ^{13}C NMR (101 MHz, CD_3OD) δ 173.20, 169.60, 167.08, 156.87, 152.17, 144.24, 143.66, 138.90, 135.20, 133.40, 133.22, 132.35, 132.26, 130.43, 128.93, 128.25, 128.11, 54.91, 52.41, 37.20, 35.87, 14.90, 14.38, 12.92, 11.57. HRMS, ESI^{+}, *m/z* calcd for C29H30N5O3S $[M+H]^+$, 528.2069; found 528.2066.

Synthesis of compound 1g

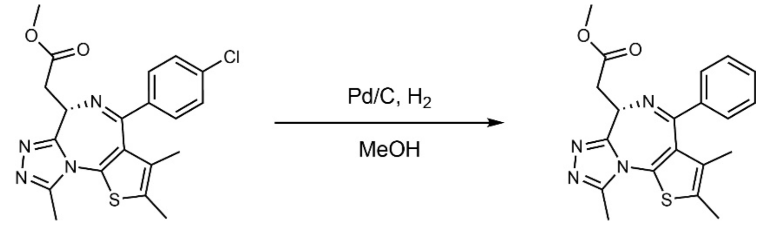

IBG1-1 **1g**

Methyl (S)-2-(2,3,9-trimethyl-4-phenyl-6H-thieno[3,2-f][1,2,4]triazolo[4,3-a][1,4]diazepin-6-yl)acetate (**1g**)

To a solution of methyl (R)-2-(4-(4-chlorophenyl)-2,3,9-trimethyl-6H-thieno[3,2-f][1,2,4]triazolo[4,3-a]azepin-6-yl)acetate (**IBG1-1**) (29.3 mg, 70.6 µmol) in MeOH (1.0 mL) was added palladium on carbon (10wt%, 7.5 mg, 7.1 µmol, 0.1 eq.) under nitrogen and then the mixture was stirred under hydrogen at room temperature overnight. The resulted mixture was diluted with excess amount of EtOAc and then stirred under air at room temperature for 1 hour. The resulted mixture was filtered, washed with EtOAc, and then concentrated by nitrogen blow. The crude mixture was suspended in EtOAc and small amount of DMSO, washed with saturated ammonium chloride aqueous solution, water, and brine, dried over $MgSO_4$, concentrated in vacuo, and then purified by silica gel column chromatography (DCM-MeOH). The obtained fraction which had the desired product was concentrated in vacuo and then purified again by preparative HPLC (ODS, H_2O-MeCN with 0.1% HCOOH) to afford **1g** (2.6 mg, 10%) as a white solid. ^{1}H NMR (500 MHz, $CDCl_3$) δ 7.48-7.42 (m, 3H), 7.38-7.35 (m, 2H), 4.66-4.64 (m, 1H), 3.80 (s, 3H), 3.69-3.66 (m, 2H), 2.70 (s, 3H), 2.43 (s, 3H), 1.69 (s, 3H), ^{13}C NMR (126 MHz, $CDCl_3$) δ 172.18, 165.02, 155.48, 149.84, 138.28, 132.10, 131.23, 130.87, 130.51, 130.35, 128.48, 128.43, 53.83, 51.83, 36.80, 14.26, 13.05, 11.83. HRMS, ESI$^+$, *m/z* calcd for C20H21N4O2S $[M+H]^+$, 381.1385; found 381.1389.

Synthesis of bIBG1

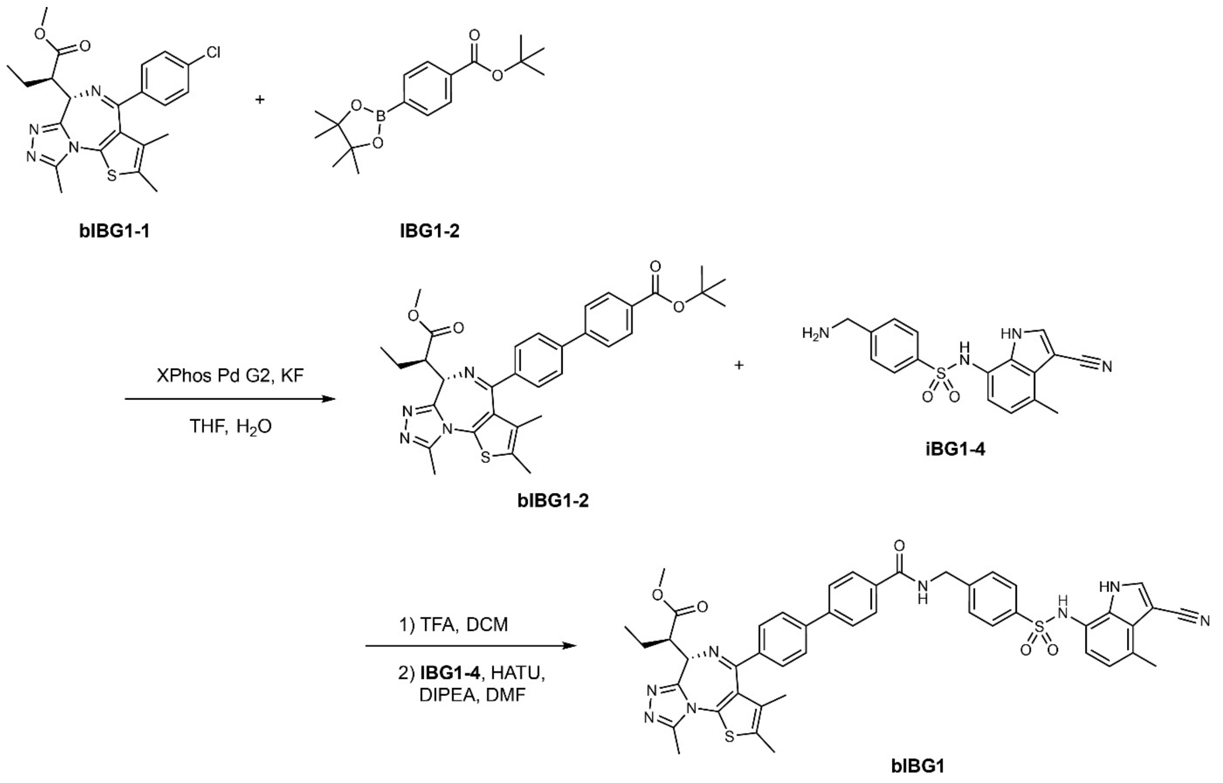

<u>tert-Butyl 4'-((S)-6-((R)-1-methoxy-1-oxobutan-2-yl)-2,3,9-trimethyl-6H-thieno[3,2-f][1,2,4]triazolo[4,3-a][1,4]diazepin-4-yl)-[1,1'-biphenyl]-4-carboxylate (**bIBG1-2**)</u>

A solution of methyl (R)-2-((S)-4-(4-chlorophenyl)-2,3,9-trimethyl-6H-thieno[3,2-f][1,2,4]triazolo[4,3-a][1,4]diazepin-6-yl)butanoate (**bIBG1-1**) (20 mg, 0.027 mmol), potassium fluoride (16 mg, 0.27 mmol, 10 eq.), 4-(tert-butoxycarbonyl)phenylboronic acid pinacol ester (24 mg, 0.080 mmol, 3.0 eq.) and [2-(2-aminophenyl)phenyl]-hydroxy-oxo-palladium [2-chloro-6-(2,4,6-triisopropylphenyl)phenyl]-dicyclohexyl-phosphane (6.6 mg, 0.0080 mmol, 0.30 eq.) in DMF (3.0 mL) and water (0.15 mL) was heated to 100 °C. After being stirred for 15 hours, the mixture was filtered and washed with water and saturated aqueous NaCl. The organic layer was extracted, dried over Na_2SO_4, filtered, and concentrated. Column chromatography of the residue (DCM-MeOH) gave the impure product, which was purified again with preparative HPLC (ODS, H_2O-MeCN with 0.1% HCOOH) to give **bIBG1-2** (9.0 mg, 0.015 mmol, 58% yield). ^1H NMR (400 MHz, CD_3OD) δ 8.05 (d, *J* = 8.4 Hz, 2H), 7.79-7.71 (m, 4H), 7.51 (d, *J* = 8.3 Hz, 2H), 4.29 (d, *J* = 11.0 Hz, 1H), 3.90 (s, 3H), 3.88 (m, 1H), 2.72 (s, 3H), 2.49 (s, 3H), 2.09 (m, 1H), 1.75 (s, 3H), 1.71 (m, 1H), 1.63 (s, 9H), 1.06 (t, *J* = 7.3 Hz, 3H). ^{13}C NMR (101 MHz, CD_3OD) δ 177.28, 167.91, 167.11, 156.70, 152.97, 146.24, 144.43, 139.68, 134.23, 134.09, 133.35, 133.18, 132.95, 131.83, 131.22, 129.18, 128.88, 83.27, 61.38, 53.10, 51.93, 29.30, 25.06, 15.26, 13.80, 12.68, 12.41. LC-MS, ESI$^+$, *m/z* 442.9 $[M+H]^+$.

methyl (R)-2-((S)-4-(4'-((4-(N-(3-Cyano-4-methyl-1H-indol-7-yl)sulfamoyl)benzyl)carbamoyl)-[1,1'-biphenyl]-4-yl)-2,3,9-trimethyl-6H-thieno[3,2-f][1,2,4]triazolo[4,3-a][1,4]diazepin-6-yl)butanoate (**bIBG1**)

To a solution of **bIBG1-2** (9.0 mg, 0.0154 mmol) in DCM (0.5 mL) was added trifluoroacetic acid (0.50 mL, 6.53 mmol). After being stirred for 3 hours, the mixture was concentrated. The residue was azeotropically dried three times with toluene. To a solution of the resultant residue and **IBG1-4** (15 mg, 0.023 mmol, 1.5 eq.) in DMF (0.8 mL) was added HATU (12 mg, 0.030 mmol, 2.0 eq.) and *N,N*-dimethylformamide (0.013 mL, 0.076 mmol, 5.0 eq.). After being stirred for 2 hours, the mixture was purified with preparative HPLC (ODS, H_2O-MeCN with 0.1% HCOOH) to give **bIBG1** (3.9 mg, 0.0046 mmol, 30% yield) .^{1}H NMR (500 MHz, CD_3OD) δ 8.00-7.95 (m, 2H), 7.92 (s, 1H), 7.83-7.34 (m, 4H), 7.69-7.62 (m, 2H), 7.56-7.45 (m, 4H), 6.75 (m, 1H), 6.52 (m, 1H), 4.66 (s, 2H), 4.30 (d, *J* = 11.0 Hz, 1H), 3.90 (s, 3H), 3.87 (dd, *J* = 10.9, 3.7 Hz, 1H), 2.73 (s, 3H), 2.66 (s, 3H), 2.50 (s, 3H), 2.10 (m, 1H), 1.77 (s, 3H), 1.71 (m, 1H), 1.06 (t, *J* = 7.45 Hz, 3H) ^{13}C NMR (126 MHz, CD_3OD) δ 177.31, 170.62, 167.24, 156.76, 153.00, 146.76, 145.43, 144.43, 139.95, 139.61, 136.36, 135.44, 134.27, 134.13, 133.17, 132.96, 131.27, 131.17, 129.96, 129.66, 129.63, 129.14, 129.10, 124.58, 122.52, 122.44, 119.37, 86.94, 61.37, 53.14, 51.92, 44.90, 25.08, 19.13, 15.28, 13.80, 12.69, 12.42. HRMS, ESI$^+$, *m/z* calcd for C46H43N8O5S2 $[M+H]^+$, 851.2798; found 851.2834.

Synthesis of IBG2

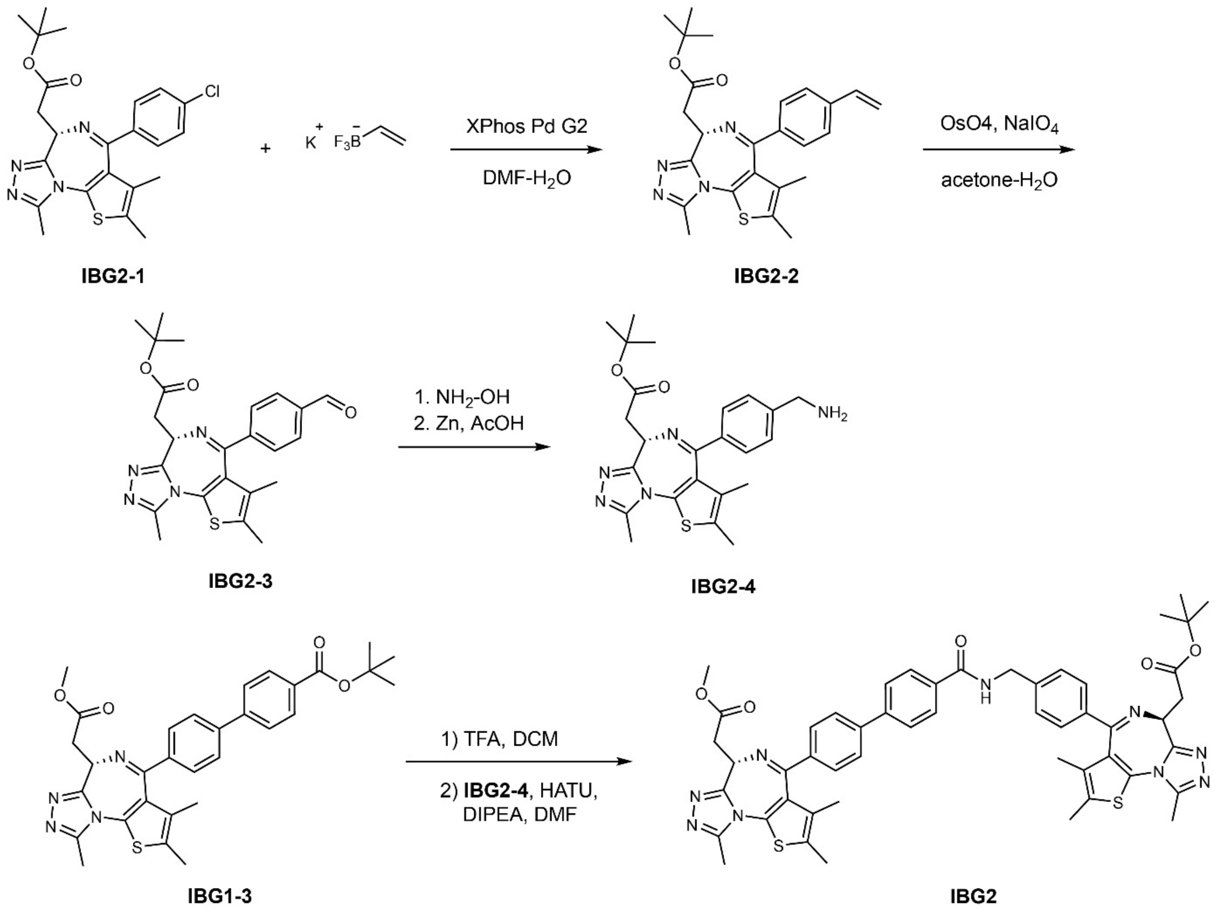

tert-Butyl (S)-2-(2,3,9-trimethyl-4-(4-vinylphenyl)-6H-thieno[3,2-f][1,2,4]triazolo[4,3-a][1,4]diazepin-6-yl)acetate (**IBG2-2**)

A solution of JQ1 (**IBG2-1**) (240 mg, 0.525 mmol), potassium vinyltrifluoroborate (211 mg, 1.58 mmol, 3.0 eq.), [2-(2-aminophenyl)phenyl]-chloro-palladium dicyclohexyl-[2-(2,4,6-triisopropylphenyl)phenyl]phosphane (124 mg, 0.158 mmol, 0.30 eq.) and *N,N*-diisopropylethylamine (0.46 mL, 2.63 mmol, 5.0 eq.) in DMF (3.0 mL) and water (0.3 mL) was heated to 130 °C. After being stirred for 15 hours, the mixture was filtered and then washed with water and saturated aqueous NaCl. The organic layer was extracted, dried over Na_2SO_4, filtered, and concentrated. Column chromatography of the residue (DCM-MeOH) gave **IBG2-2** (180 mg, 0.40 mmol, 76% yield). ^{1}H NMR (500 MHz, $CDCl_3$) δ 7.42 (d, *J* = 8.3 Hz, 2H), 7.38 (d, *J* = 8.6 Hz, 2H), 6.71 (dd, *J* = 17.6, 10.9 Hz, 1H), 5.80 (d, *J* = 17.6 Hz, 1H), 5.31 (d, *J* = 11.0 Hz, 1H), 4.56 (dd, *J* = 7.2, 6.9 Hz, 1H), 3.55 (d, *J* = 6.9 Hz, 2H), 2.67 (s, 3H), 2.40 (s, 3H), 1.69 (s, 3H), 1.50 (s, 9H). ^{13}C NMR (126 MHz, $CDCl_3$) δ 171.00, 164.41, 155.77, 149.85, 139.71, 137.69, 136.25, 132.15, 131.21, 130.94, 130.43, 128.82, 126.34, 115.46, 80.94, 54.00, 38.05, 28.30, 14.48, 13.20, 12.00. LC-MS, ESI$^-$, *m/z* 449.1 $[M+H]^+$.

tert-Butyl (S)-2-(4-(4-formylphenyl)-2,3,9-trimethyl-6H-thieno[3,2-f][1,2,4]triazolo[4,3-a][1,4]diazepin-6-yl)acetate (**IBG2-3**)

To a solution of **IBG2-2** (200 mg, 0.446 mmol, 1.0 eq.) and sodium periodate (286 mg, 1.34 mmol, 3.0 eq.) in acetone (5.0 mL) and water (1.0 mL) was added osmium tetroxide (0.14 mL as a 4% aqueous solution, 0.0223 mmol, 0.050 eq.). The reaction was stirred for 2 hours, and then diluted with EtOAc. The organic layer was washed with water and saturated aqueous NaCl, dried over Na_2SO_4, filtered, and concentrated. Column chromatography of the residue (DCM-MeOH) to give **IBG2-3** (180 mg,0.40 mmol, 90% yield). ^{1}H NMR (500 MHz, $CDCl_3$) δ 10.04 (s, 1H), 7.87 (d, *J* = 8.4, 2H), 7.63 (d, *J* = 8.1 Hz, 2H), 4.61 (dd, *J* = 7.0, 7.0 Hz, 1H), 3.57 (d, *J* = 7.0 Hz, 2H), 2.69 (s, 3H), 2.41 (s, 3H), 1.66 (s, 3H), 1.51 (s, 9H). ^{13}C NMR (126 MHz, $CDCl_3$) δ 191.78, 170.87, 163.96, 155.35, 149.99, 143.68, 137.51, 132.63, 131.00, 130.72, 130.22, 129.82, 129.24, 81.12, 54.30, 37.91, 28.29, 14.48, 13.22, 12.01. LC-MS, ESI$^-$, *m/z* 451.2 $[M+H]^+$.

tert-Butyl (S)-2-(4-(4-(aminomethyl)phenyl)-2,3,9-trimethyl-6H-thieno[3,2-f][1,2,4]triazolo[4,3-a][1,4]diazepin-6-yl)acetate (**IBG2-4**)

To a solution of **IBG2-3** (30 mg, 0.0666 mmol) in ethanol (1.0 mL) was added hydroxylamine hydrochloride (16 mg, 0.226 mmol, 3.4 eq.) and sodium acetate (22 mg, 0.266 mmol, 4.0 eq.). The reaction mixture was stirred at 70 °C for 3 hours. The mixture was cooled to room temperature and diluted with EtOAc, and then quenched with water. The organic layer was washed with saturated aqueous NaCl, dried over Na_2SO_4, filtered, and concentrated. The crude was used in the next reaction without further purifications. LC-MS, ESI$^-$, *m/z* 446.2 $[M+H]^+$.

A mixture of the resultant crude and zinc (8.4 mg, 0.129 mmol) were dissolved in acetic acid (1.0 mL) and stirred at 40 °C. After being stirred for 20 hours, another 5 mg of zinc was added. The reaction was stirred for 2 hours, and then added another 1 mg of zinc. After being stirred for additional 3 hours, the reaction was diluted with DCM and filtered. The filtrate was concentrated and purified with preparative HPLC (ODS, H_2O-MeCN with 0.1% HCOOH) to give **IBG2-4** (13 mg, 0.0288 mmol, 45% yield). ^{1}H NMR (500 MHz, CD_3OD) δ 7.46 (d, *J* = 8.3 Hz, 2H), 7.41 (d, *J* = 8.4 Hz, 2H), 4.58 (dd, *J* = 8.7, 5.8 Hz, 1H), 3.88 (brs, 2H), 3.53-3.38 (m, 2H), 2.73 (s, 3H), 2.48 (s, 3H), 1.72 (s, 3H), 1.53 (s, 9H). ^{13}C NMR (126 MHz, CD_3OD) δ 172.73, 168.02, 157.79, 152.90, 147.09, 138.92, 134.13, 133.95, 133.28, 133.04, 130.79, 129.50, 83.19, 55.85, 47.01, 39.34, 29.26, 15.17, 13.78, 12.42. LCMS was only detected as sulfonic acid form: LC-MS, ESI$^-$, *m/z* 452.10 $[M+H]^+$.

tert-Butyl 2-((S)-4-(4-((4'-((S)-6-(2-(tert-butoxy)-2-oxoethyl)-2,3,9-trimethyl-6H-thieno[3,2-f][1,2,4]triazolo[4,3-a][1,4]diazepin-4-yl)-[1,1'-biphenyl]-4-carboxamido)methyl)phenyl)-2,3,9-trimethyl-6H-thieno[3,2-f][1,2,4]triazolo[4,3-a][1,4]diazepin-6-yl)acetate (**IBG2**)

To a solution of **IBG1-3** (10.0 mg, 0.018 mmol) in DCM (0.5 mL) was added trifluoroacetic acid (0.5 mL), and then the mixture was stirred at room temperature for 5 hours. The reaction mixture was concentrated, and the residue was azeotropically dried

three times with toluene. To a solution of the resultant crude and **IBG2-4** (5.4 mg, 0.012 mmol) in DMF (0.5 mL) was added HATU (9.1 mg, 0.0240 mmol, 2.0 eq.) and *N,N*-diisopropylethylamine (0.010 mL, 0.0599 mmol, 5.0 eq.). After being stirred for 3 hours, the mixture was diluted with EtOAc, and then quenched with aqueous $NaHCO_3$. The mixture was extracted with EtOAc, and the organic layer was washed with saturated aqueous NaCl, dried over Na_2SO_4, filtered, and concentrated. The residue was purified with preparative HPLC (ODS, H_2O-MeCN with 0.1% HCOOH) to give **IBG2** (6.0 mg, 0.00642 mmol, 54% yield). ^1H NMR (500 MHz, CD_3OD) δ 7.97 (d, *J* = 8.4 Hz, 2H), 7.79 (d, *J* = 8.4 Hz, 2H), 7.75 (d, *J* = 8.5 Hz, 2H), 7.58 (d, *J* = 8.1 Hz, 2H), 7.49-7.41 (m, 4H), 4.70-464 (m, 3H), 4.57 (dd, *J* = 8.6, 5.9 Hz, 1H), 3.80 (s, 3H), 3.59 (d, *J* = 7.5 Hz, 2H), 3.44 (m, 2H), 2.73 (s, 3H), 2.71 (s, 3H), 2.49 (s, 3H), 2.46 (s, 3H), 1.76 (s, 3H), 1.70 (s, 3H), 1.51 (s. 9H). ^{13}C NMR (126 MHz, CD_3OD) δ 174.02, 172.75, 170.52, 168.00, 167.91, 157.76, 157.67, 153.04, 152.92, 145.30, 144.37, 144.28, 139.76, 139.08, 135.57, 134.24, 134.14, 134.07, 133.98, 133.22, 133.12, 133.07, 133.04, 131.30, 130.79, 129.95, 129.47, 129.11, 129.04, 83.23, 55.86, 55.72, 53.31, 45.04, 39.34, 38.02, 29.26, 15.29, 15.22, 13.81, 13.79, 12.47, 12.43. HRMS, ESI+, m/z calcd for C51H52N9O5S2 $[M+H]^+$, 934.353; found 934.359.

Synthesis of IBG3

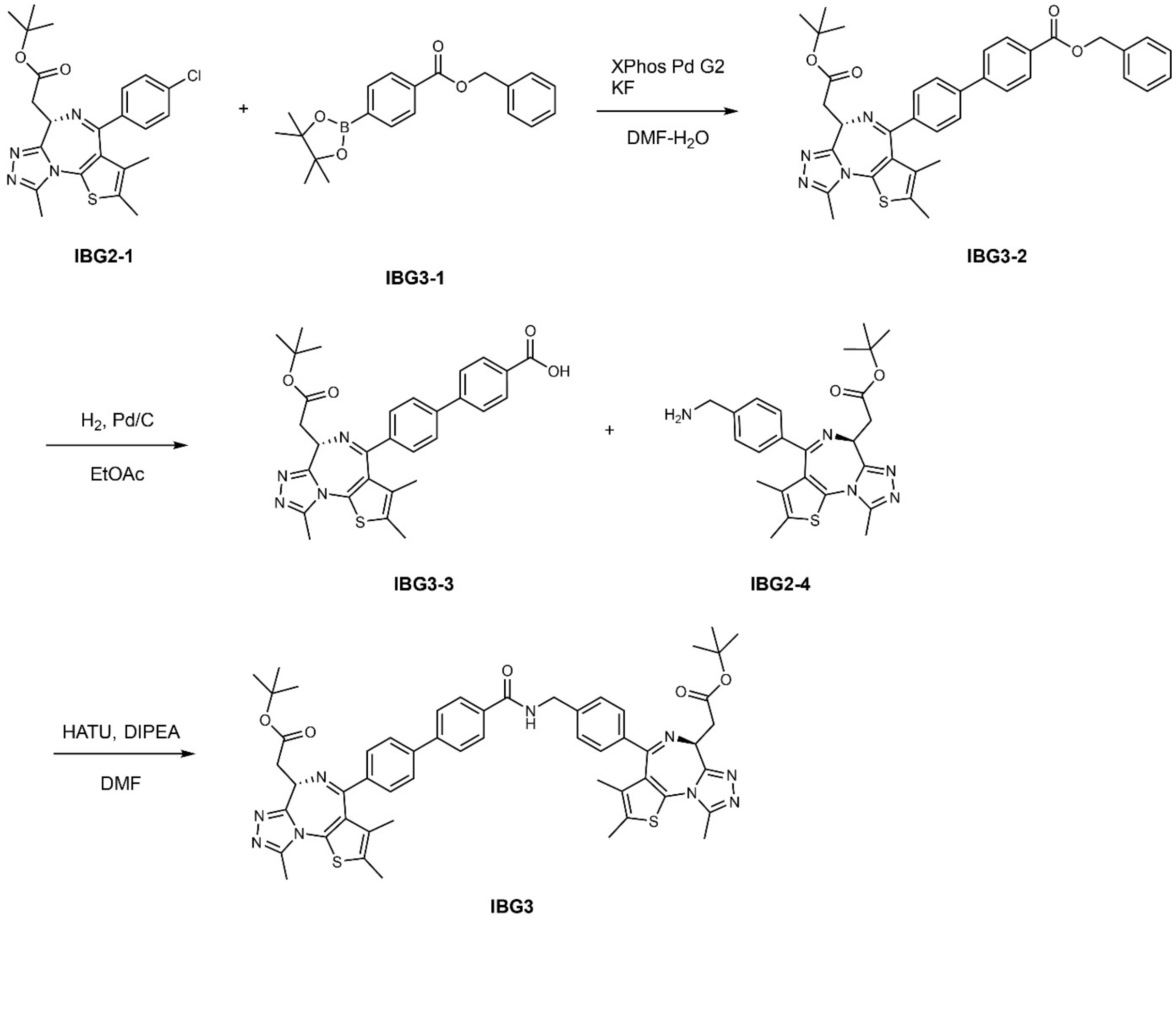

Benzyl (S)-4'-(6-(2-(tert-butoxy)-2-oxoethyl)-2,3,9-trimethyl-6H-thieno[3,2-f][1,2,4]triazolo[4,3-a][1,4]diazepin-4-yl)-[1,1'-biphenyl]-4-carboxylate (**IBG3-2**)

A solution of tert-butyl 2-[rac-(9S)-7-(4-chlorophenyl)-4,5,13-trimethyl-3-thia-1,8,11,12-tetrazatricyclo[8.3.0.02,6]trideca-2(6),4,7,10,12-pentaen-9-yl]acetate JQ1 (**IBG2-1**) (50 mg, 0.109 mmol, 1.0 eq.), benzyl 4-(4,4,5,5-tetramethyl-1,3,2-dioxaborolan-2-yl)benzoate (**IBG3-1**) (66.6 mg, 0.197 mmol, 1.8 eq.), [2-(2-aminophenyl)phenyl]-hydroxy-oxo-palladium [2-chloro-6-(2,4,6-triisopropylphenyl)phenyl]-dicyclohexyl-phosphane (27 mg, 0.0328 mmol, 0.3 eq.), and potassium fluoride (32 mg, 0.547 mmol, 5.0 eq.) in DMF (3.0 mL) and water (0.15 mL) was heated to 130 °C. After being stirred for 15 hours, the mixture was filtered and then, washed with water and saturated aqueous NaCl. The organic layer was extracted, dried over Na_2SO_4, filtered, and concentrated. Column chromatography of the residue (DCM-MeOH) gave impure product, which was purified with preparative HPLC (ODS, H_2O-MeCN with 0.1% HCOOH) gave **IBG3-2** (38 mg, 0.060 mmol, 55% yield). ^{1}H NMR (500 MHz, $CDCl_3$) δ 8.11 (m, 2H), 7.75 (m, 4H), 7.57 (d, *J* = 7.6 Hz, 2H), 7.48 (d, *J* = 7.8 Hz, 2H), 7.43-7.32 (m, 3H), 5.38 (s, 2H), 4.59 (dd, *J* = 8.7, 5.8 Hz, 1H), 3.54-3.40 (m, 2H), 2.72 (s, 3H), 2.46 (s, 3H), 1.74 (s, 3H), 1.53 (s, 9H). ^{13}C NMR (126 MHz, $CDCl_3$) δ 170.51, 166.10, 165.37, 155.47, 150.67, 144.44, 141.94, 137.65, 136.17, 131.98, 131.81, 130.85, 130.71, 129.85, 129.26, 129.00, 128.24, 128.12, 128.02, 127.93, 127.85, 126.96, 126.82, 80.98, 53.72, 37.16, 13.05, 11.58, 10.22. LC-MS, ESI^-, *m/z* 633.2 $[M+H]^+$.

(S)-4'-(6-(2-(tert-butoxy)-2-oxoethyl)-2,3,9-trimethyl-6H-thieno[3,2-f][1,2,4]triazolo[4,3-a][1,4]diazepin-4-yl)-[1,1'-biphenyl]-4-carboxylic acid (**IBG3-3**)

To a solution of **IBG3-2** (38 mg, 0.0601 mmol) in EtOAc (1.0 mL) was added palladium on carbon (5%, 6.4 mg, 0.05 eq.), and the mixture was stirred under hydrogen atmosphere at room temperature for 5 hours. The suspension was filtered through a pad of silica gel with 20% MeOH in DCM. Without further purifications the crude was used in the next reaction. LC-MS, ESI^-, *m/z* 543.2 $[M+H]^+$.

tert-Butyl 2-((S)-4-(4-((4'-((S)-6-(2-(tert-butoxy)-2-oxoethyl)-2,3,9-trimethyl-6H-thieno[3,2-f][1,2,4]triazolo[4,3-a][1,4]diazepin-4-yl)-[1,1'-biphenyl]-4-carboxamido)methyl)phenyl)-2,3,9-trimethyl-6H-thieno[3,2-f][1,2,4]triazolo[4,3-a][1,4]diazepin-6-yl)acetate (**IBG3**)

To a solution of **IBG2-4** (13 mg, 0.0288 mmol) and **IBG3-3** (17 mg, 0.0313 mmol, 1.1 eq.) in DMF (0.5 mL) was added HATU (22 mg, 0.0576 mmol, 2.0 eq.) and *N,N*-diisopropylethylamine (0.025 mL, 0.144 mmol, 5.0 eq.). After being stirred for 3 hours, the mixture was diluted with EtOAc, and then quenched with aqueous $NaHCO_3$. The mixture was extracted with EtOAc, and the organic layer was washed with saturated aqueous NH4Cl and NaCl, dried over Na_2SO_4, filtered, and concentrated. The residue was purified with preparative HPLC (ODS, H_2O-MeCN with 0.1% HCOOH) to give **IBG3** (10.0 mg, 0.0102 mmol, 36% yield). ^{1}H NMR (500 MHz, CDl_3OD) δ 8.00-7.94 (m, 2H), 7.82-7.72 (m, 4H), 7.62-7.56 (m, 2H), 7.49-7.40 (m, 4H), 4.66 (brs, 2H), 4.61 (dd, *J* = 8.6, 5.8 Hz, 1H), 4.56 (dd, *J* = 8.6, 5.9 Hz, 1H), 3.67-3.55 (m, 4H), 2.73 (s, 3H), 2.71 (s, 3H), 2.49 (s, 3H), 2.46 (s, 3H), 1.76, (s, 3H), 1.70 (s, 3H), 1.54 (s, 9H), 1.51 (s, 9H). ^{13}C NMR (126 MHz, CD_3OD) δ 172.74, 172.72, 170.50, 167.95, 167.70,

157.77, 152.94, 145.28, 144.38, 144.28, 139.74, 139.09, 135.58, 134.25, 134.14, 134.06, 133.95, 133.23, 133.16, 133.04, 132.99, 131.25, 130.79, 129.94, 129.45, 129.11, 129.04, 83.20, 83.18, 55.97, 55.86, 45.04, 39.40, 39.35, 29.28, 29.26, 15.28, 15.22, 13.81, 13.78, 12.45, 12.42. HRMS, ESI+, m/z calcd for C54H58N9O5S2 $[M+H]^+$, 976.4002; found 976.4047.

Synthesis of IBG4

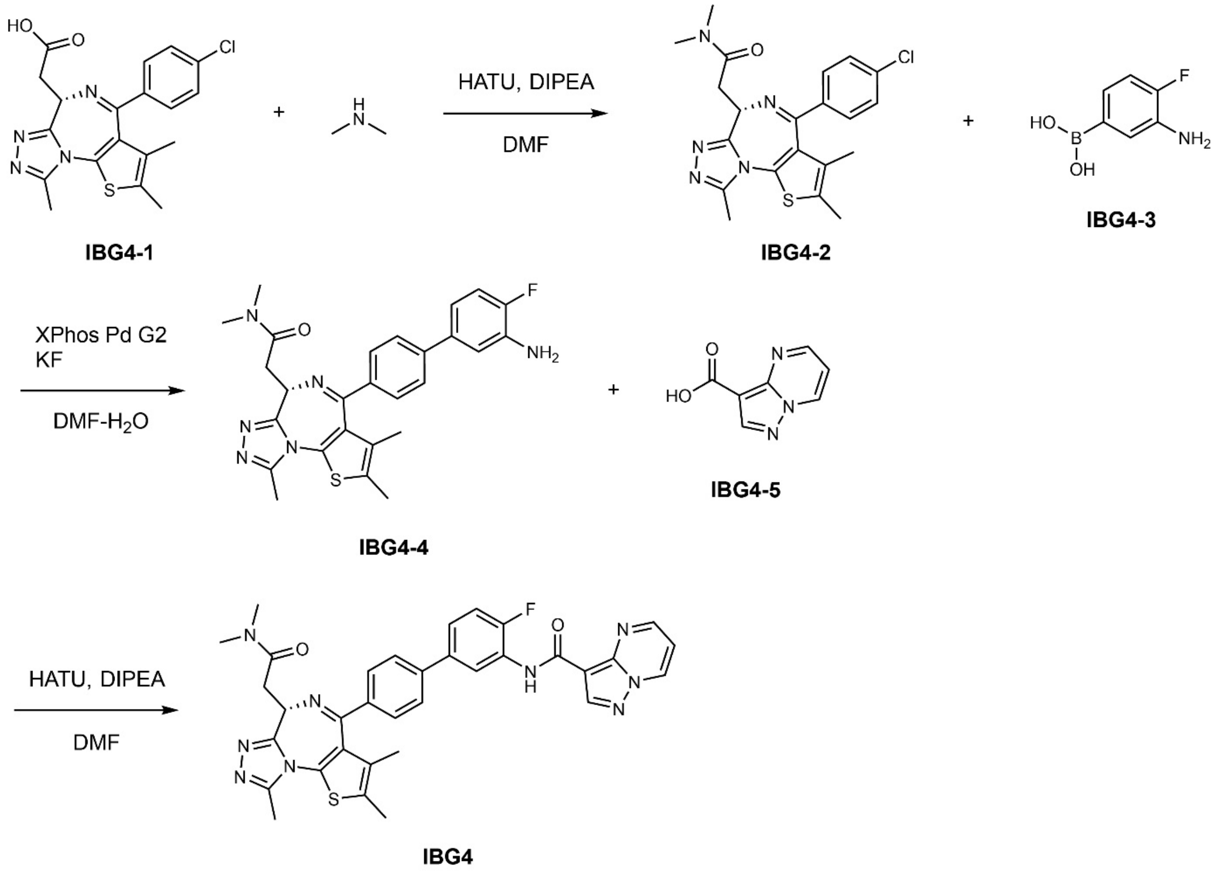

(S)-2-(4-(4-Chlorophenyl)-2,3,9-trimethyl-6H-thieno[3,2-f][1,2,4]triazolo[4,3-a][1,4]diazepin-6-yl)-N,N-dimethylacetamide (**IBG4-2**)

To a solution of (S)-2-(4-(4-chlorophenyl)-2,3,9-trimethyl-6H-thieno[3,2-f][1,2,4]triazolo[4,3-a][1,4]diazepin-6-yl)acetic acid (**IBG4-1**) (87 mg, 0.218 mmol) in DMF (0.5 mL) was added *N,N*-diisopropylethylamine (0.19 mL, 1.09 mmol, 5.0 eq.), dimethylamine (2 M in THF, 1.1 mL, 2.18 mmol, 10 eq.), and HATU (166 mg, 0.436 mmol, 2.0 eq.). After being stirred at room temperature for 3 hours, the mixture was diluted with EtOAc, and then quenched with aqueous $NaHCO_3$. The mixture was extracted with EtOAc, and the organic layer was washed with saturated aqueous NaCl, dried over Na_2SO_4, filtered, and concentrated. Preparative HPLC purification (ODS, H_2O-MeCN with 0.1% HCOOH) of the residue gave **IBG4-2** (75 mg, 0.175 mmol, 80% yield). ^1H NMR (500 MHz, $CDCl_3$) δ 7.39 (d, J = 8.5 Hz, 2H), 7.31 (d, J = 8.7 Hz, 2H),

4.79 (dd, *J* = 7.4, 6.0 Hz, 1H), 3.66 (dd, *J* = 16.1, 5.1 Hz, 1H), 3.58 (dd, *J* = 16.1, 7.5 Hz, 1H), 3.24 (s, 3H), 3.00 (s, 3H), 2.66 (s, 3H), 2.38 (s, 3H), 1.66 (s, 3H). ^{13}C NMR (126 MHz, $CDCl_3$) δ 170.60, 163.70, 156.08, 149.88, 136.98, 136.68, 132.31, 131.03, 130.69, 129.94, 128.76, 54.62, 37.67, 35.69, 35.63, 14.48, 13.19, 11.96. LC-MS, ESI$^-$, *m/z* 428.1 $[M+H]^+$.

(S)-2-(4-(3'-amino-4'-fluoro-[1,1'-biphenyl]-4-yl)-2,3,9-trimethyl-6H-thieno[3,2-f][1,2,4]triazolo[4,3-a][1,4]diazepin-6-yl)-N,N-dimethylacetamide (**IBG4-4**)

A mixture of **IBG4-2** (38 mg, 0.0876 mmol), (3-amino-4-fluoro-phenyl)boronic acid (**IBG4-3**) (41 mg, 0.263 mmol, 3.0 eq.), potassium fluoride (25 mg, 0.438 mmol, 5.0 eq.), and [2-(2-aminophenyl)phenyl]-chloro-palladium;dicyclohexyl-[2-(2,4,6-triisopropylphenyl)phenyl]phosphane (21 mg, 0.0263 mmol, 0.3 eq.) was dissolved in DMF (1.0 mL) and water (0.15 mL). After being stirred at 130 °C for 20 hours, the reaction was cooled to room temperature, diluted with EtOAc and then quenched with saturated aqueous $NaHCO_3$. The mixture was extracted with EtOAc, and the organic layer was washed with saturated aqueous NaCl, dried over Na_2SO_4, filtered, and concentrated. Column chromatography (ODS, H_2O-MeCN with 0.1% HCOOH) of the residue gave the product as a salt of formic acid. The product was dissolved in EtOAc. and washed with saturated aqueous $NaHCO_3$, dried over Na_2SO_4, filtered, and concentrated to give **IBG4-4** (20 mg, 0.0398 mmol, 45% yield). ^{1}H NMR (500 MHz, $CDCl_3$) δ 7.48 (brs, 4H), 7.05-6.95 (m, 2H), 6.89 (m, 1H), 4.82 (dd, *J* = 6.1, 1.2 Hz, 1H), 3.82 (brs, 2H), 3.67 (dd, *J* = 9.9, 6.1 Hz, 1H), 3.60 (dd, *J* = 8.6, 7.4 Hz, 1H), 3.26 (s, 3H), 3.01 (s, 3H), 2.66 (s, 3H), 2.69 (s, 3H), 1.70 (s, 3H). ^{13}C NMR (126 MHz, $CDCl_3$) δ 170.73, 164.48, 156.22, 151.73 (d, *J* = 240.2 Hz), 149.83, 142.70, 137.30, 136.95 (d, *J* = 3.1 Hz), 134.87 (d, *J* = 13.3 Hz), 132.13, 131.30, 131.09, 130.39, 128.96, 126.98 117.47 (d, *J* = 6.7 Hz), 115.66 (d, *J* = 14.9 Hz), 115.54 (d, *J* = 7.9 Hz), 54.64, 37.69, 35.71, 35.68, 14.51, 13.18, 11.97. LC-MS, ESI$^-$, *m/z* 503.1 $[M+H]^+$.

(S)-N-(4'-(6-(2-(Dimethylamino)-2-oxoethyl)-2,3,9-trimethyl-6H-thieno[3,2-f][1,2,4]triazolo[4,3-a][1,4]diazepin-4-yl)-4-fluoro-[1,1'-biphenyl]-3-yl)pyrazolo[1,5-a]pyrimidine-3-carboxamide (**IBG4**)

To a solution of **IBG4-4** (20 mg, 0.0398 mmol), pyrazolo[1,5-a]pyrimidine-3-carboxylic acid (**IBG4-5**) (13 mg, 0.0796 mmol, 2.0 eq.), and HATU (30 mg, 0.0796 mmol, 2.0 eq.) in DMF (0.5 mL) was added *N,N*-diisopropylethylamine (0.035 mL, 0.199 mmol, 5.0 eq.) and 4-(dimethylamino)pyridin (24 mg, 0.199 mmol, 5.0 eq.). After being stirred at room temperature for 2 hours, another pyrazolo[1,5-a]pyrimidine-3-carboxylic acid (13 mg, 0.0796 mmol, 2.0 eq.), HATU (30 mg, 0.0796 mmol, 2.0 eq.), and *N,N*-diisopropylethylamine (0.035 mL, 0.199 mmol, 5.0 eq.) were added. The reaction was stirred for additional 90 hours at room temperature. The reaction was diluted with EtOAc and quenched with saturated aqueous $NaHCO_3$. The organic layer was dried over Na_2SO_4, filtered, and concentrated. Preparative HPLC purification (ODS, H_2O-MeCN with 0.1% HCOOH) of the residue gave **IBG4** (2.5 mg, 0.00389 mmol, 10% yield). ^{1}H NMR (500 MHz, CD_3OD) δ 9.15 (dd, *J* = 5.4, 1.6 Hz, 1H), 8.89 (dd, *J* = 2.6, 1.6 Hz, 1H), 8.80 (dd, *J* = 5.1, 2.3 Hz, 1H), 8.70 (s, 1H), 7.72 (d, *J* = 8.6 Hz, 2H), 7.57 (d, *J* = 8.3 Hz, 2H), 7.45 (m, 1H), 7.33 (dd, *J* = 10.7, 8.6 Hz, 1H), 7.30 (dd, *J* = 4.2, 2.8 Hz, 1H), 4.74 (dd, *J* = 7.4, 6.5 Hz, 1H), 3.69 (dd, *J* = 16.4, 7.5 Hz, 1H), 3.63 (dd, *J* = 16.4, 6.4 Hz, 1H), 3.33 (s, 3H), 3.06 (s, 3H), 2.76 (s, 3H), 2.50 (s, 3H), 1.78 (s. 3H).

^{13}C NMR (126 MHz, CD_3OD) δ 173.27, 167.89, 163.19, 158.24, 155.06, 154.82 (d, *J* = 245 Hz), 152.97, 152.50, 148.38, 148.27, 144.69, 139.55, 139.36, 138.84, 134.20, 133.94, 133.27 (d, *J* = 77.8 Hz), 131.25, 129.20 (d, *J* = 10.6 Hz), 128.94, 124.95 (d, *J* = 7.7 Hz), 122.44, 117.38 (d, *J* = 19.7 Hz), 112.32, 107.02, 56.33, 38.68, 37.05, 36.70, 15.26, 13.80, 12.47. HRMS, ESI+, m/z calcd for C34H31N9O2SF [M+H]+, 648.2305; found 648.2334.

Synthesis of DAT389

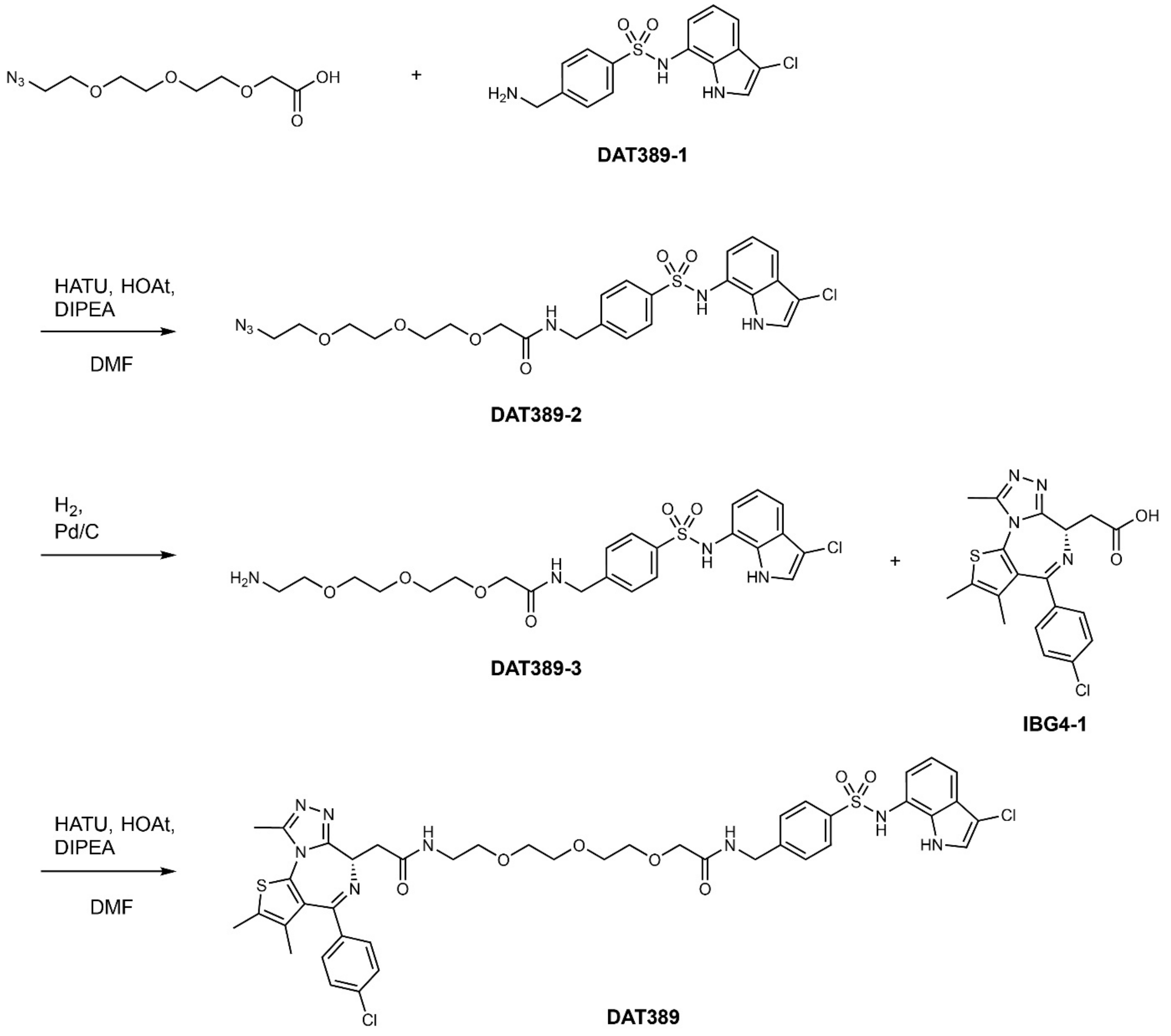

<u>2-(2-(2-(2-Azidoethoxy)ethoxy)ethoxy)-*N*-(4-(*N*-(3-chloro-1H-indol-7-yl)sulfamoyl)benzyl)acetamide (**DAT389-2**)</u>

To a solution of 4-(aminomethyl)-*N*-(3-chloro-1H-indol-7-yl)benzenesulfonamide (19.7 mg, 0.059 mmol), 2-[2-[2-(2-azidoethoxy)ethoxy]ethoxy]acetic acid (16.4 mg, 0.070 mmol, 1.2 eq.) (**DAT389-1**)[10] *N,N*-diisopropylethylamine (50 µl, 0.29 mmol, 5.0 eq.)

and 1-hydroxy-7-azabenzotriazole (6.3 mg, 0.046 mmol, 0.8 eq.) in DMF (0.5 mL) was added HATU (24.5 mg, 0.065 mmol, 1.1 eq.) at room temperature. The reaction was stirred for 1 hour. The crude mixture was then purified with reverse-phase column chromatography (0.1% formic acid in acetonitrile-water) to give **DAT389-2** (19.0 mg, 0.035 mmol, 59% yield). ^{1}H NMR (400 MHz, CD_3OD) δ 7.70-7.65 (m, 2H), 7.43-7.37 (m, 2H), 7.34 (dd, *J* = 8.0, 1.0 Hz, 1H), 7.27 (s, 1H), 6.92 (dd, *J* = 7.6, 7.6 Hz, 1H), 6.71 (dd, *J* = 7.6, 0.8 Hz, 1H), 4.50 (s, 2H), 4.08 (s, 2H), 3.74-3.66 (m, 4H), 3.63-3.57 (m, 2H), 3.57-3.52 (m, 4H), 3.30 (t, *J* = 5.1 Hz, 2H). ^{13}C NMR (101 MHz, CD_3OD) δ 173.09, 145.37, 139.50, 131.96, 128.78, 128.63, 1128.25, 123.24, 123.18, 120.95, 119.10, 116.78, 106.29, 72.05, 71.44, 71.42, 71.36, 71.23, 70.97, 51.68, 42.94. LC-MS, ESI$^+$, *m/z* 551.2 $[M+H]^+$.

2-(2-(2-(2-Aminoethoxy)ethoxy)ethoxy)-*N*-(4-(*N*-(3-chloro-1H-indol-7-yl)sulfamoyl)benzyl)acetamide (**DAT389-3**)

To a solution of **DAT-389-2** (19.0 mg, 0.035 mmol) in MeOH (3.0 mL) was added 10% Pd/C (2.0 mg). The reaction mixture was degassed under reduced pressure and filled with H_2 gas. After being stirred at room temperature for 2 hours, the mixture was filtered, and concentrated. The product was used in the next reaction without further purifications.

(S)-*N*-(4-(*N*-(3-Chloro-1H-indol-7-yl)sulfamoyl)benzyl)-2-(2-(2-(2-(2-(4-(4-chlorophenyl)-2,3,9-trimethyl-6H-thieno[3,2-f][1,2,4]triazolo[4,3-a][1,4]diazepin-6-yl)acetamido)ethoxy)ethoxy)ethoxy)acetamide (**DAT389**)

To a solution of the obtained crude **DAT389-3** (0.035 mmol), (S)-2-(4-(4-chlorophenyl)-2,3,9-trimethyl-6H-thieno[3,2-f][1,2,4]triazolo[4,3-a][1,4]diazepin-6-yl)acetic acid (14.0 mg, 0.035 mmol, 1.0 eq.), *N,N*-diisopropylethylamine (13.5 µl, 0.10 mmol, 3.0 eq.), and 1-hydroxy-7-azabenzotriazole (4.6 mg, 0.035 mmol, 1.0 eq.) in DMF (0.5 mL) was added HATU (13 mg, 0.035 mmol, 1.0 eq.) at room temperature. The reaction was allowed to stir for 1 hour. Then the mixture was purified with reverse-phase preparative HPLC chromatography (0.1% formic acid in acetonitrile-water) to give **DAT389** (10.1 mg, 0.011 mmol, 32% yield). ^{1}H NMR (400 MHz, CD_3OD) δ 7.58-7.57 (m, 2H), 7.46-7.38 (m, 4H), 7.33-7.29 (m, 3H), 7.25 (s, 1H), 6.89 (t, *J* = 7.7 Hz, 1H), 6.70 (dd, *J* = 7.53, 0.77 Hz, 1H), 4.66 (dd, *J* = 8.8, 5.5 Hz, 1H), 4.45 (s, 2H), 4.08 (s, 2H), 3.74-3.67 (m, 4H), 3.64-3.61 (m, 2H), 3.60-3.53 (m, 4H), 3.49-3.30 (m, 4H), 2.69 (s, 3H), 2.46 (s, 3H), 1.69 (s, 3H). ^{13}C NMR (106 MHz, CD_3OD) δ 173.9, 167.1, 157.9, 153.1, 146.2, 140.5, 139.0, 138.9, 134.4, 134.1, 132.92, 132.89, 132.8, 132.2, 130.7, 129.7, 129.4, 129.1, 124.3, 124.1, 121.9, 119.9, 117.5, 107.2, 72.9, 72.4, 72.3, 72.2, 72.1, 71.5, 56.1, 43.9, 41.4, 39.7, 15.3, 13.8, 12.5. LC-MS, ESI$^+$, *m/z* 906.9 $[M+H]^+$.

Supplementary Figure 1 | Uncropped western blots and SDS-PAGE images.

Figure 1b.

Solid boxes indicate regions shown in the figure, dashed box indicates region not shown in manuscript but quantified in Extended Data Figure 1e. Asterisks indicate unspecific bands.

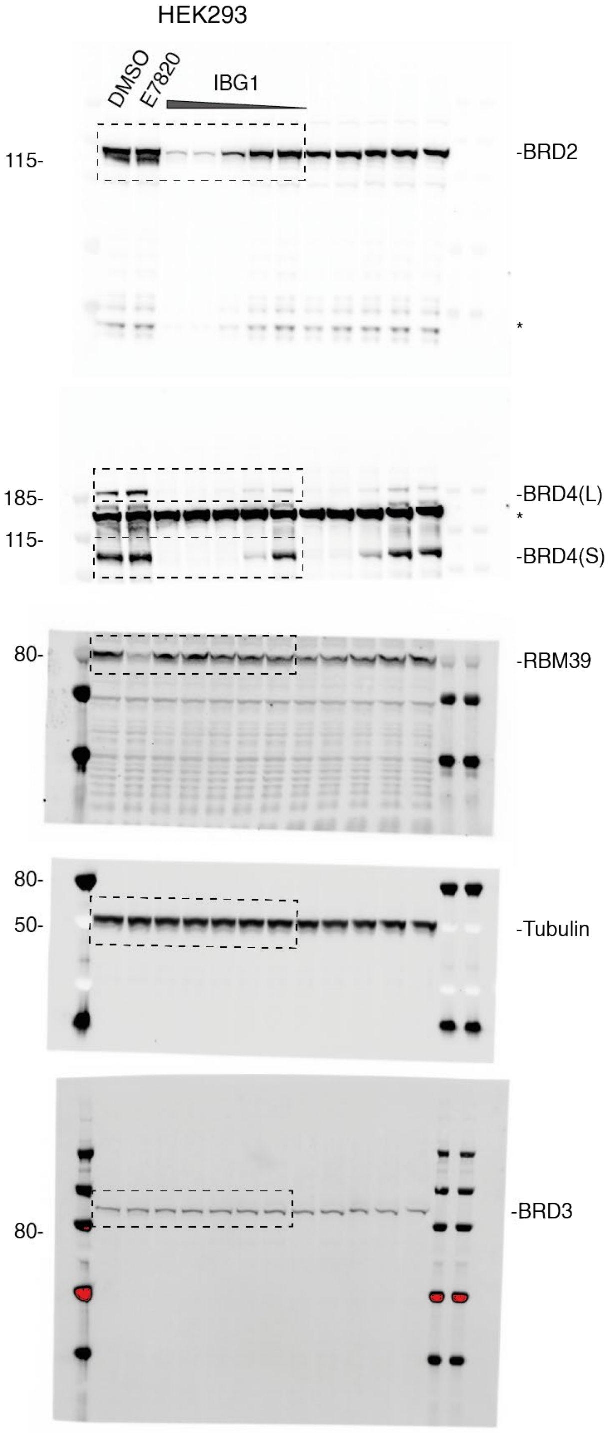

Figure 1f.

Boxes indicate regions shown in the figure. Asterisks indicate unspecific bands.

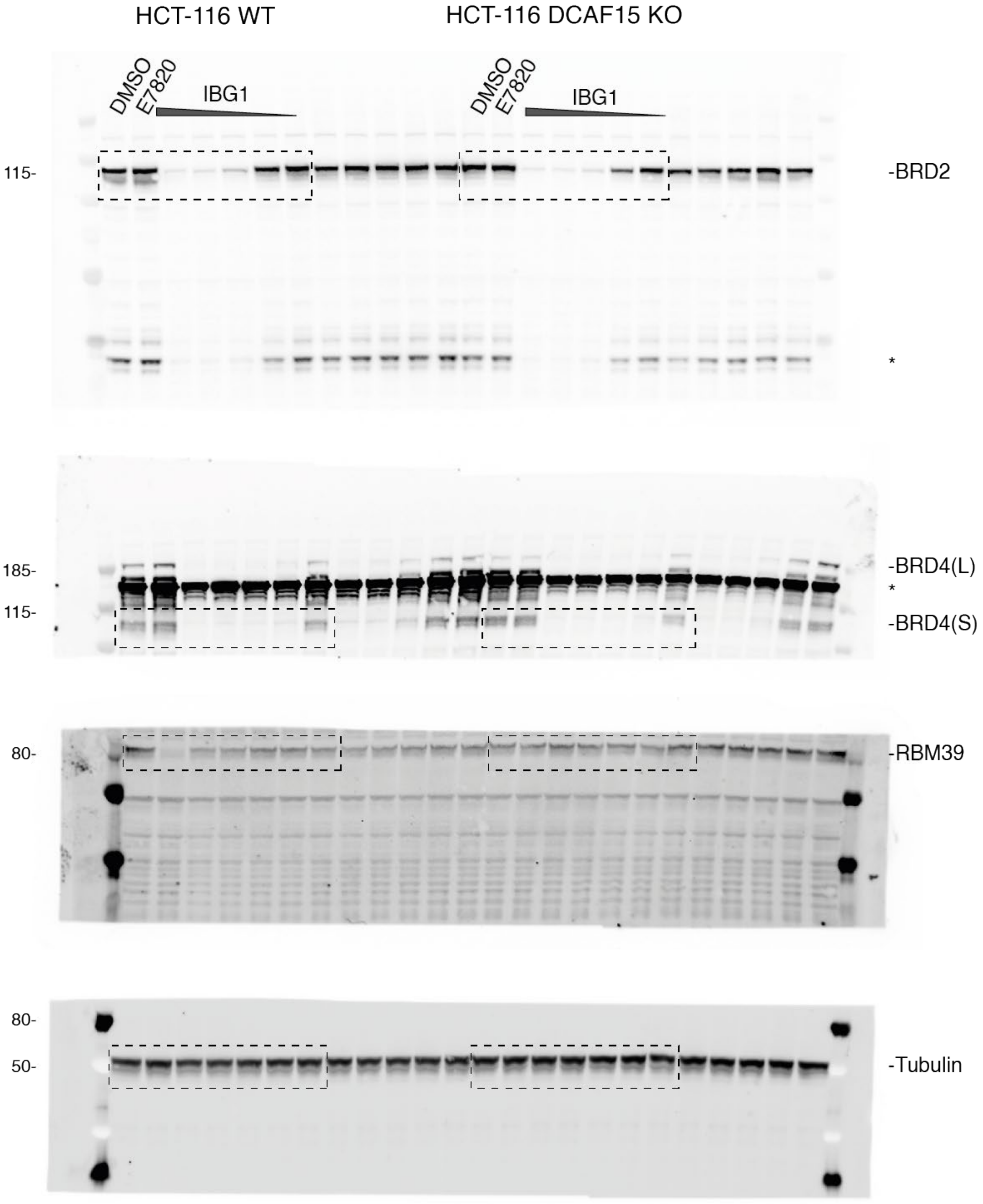

Figure 5d.

Replicate 1 | Boxes indicate regions shown in the figure.

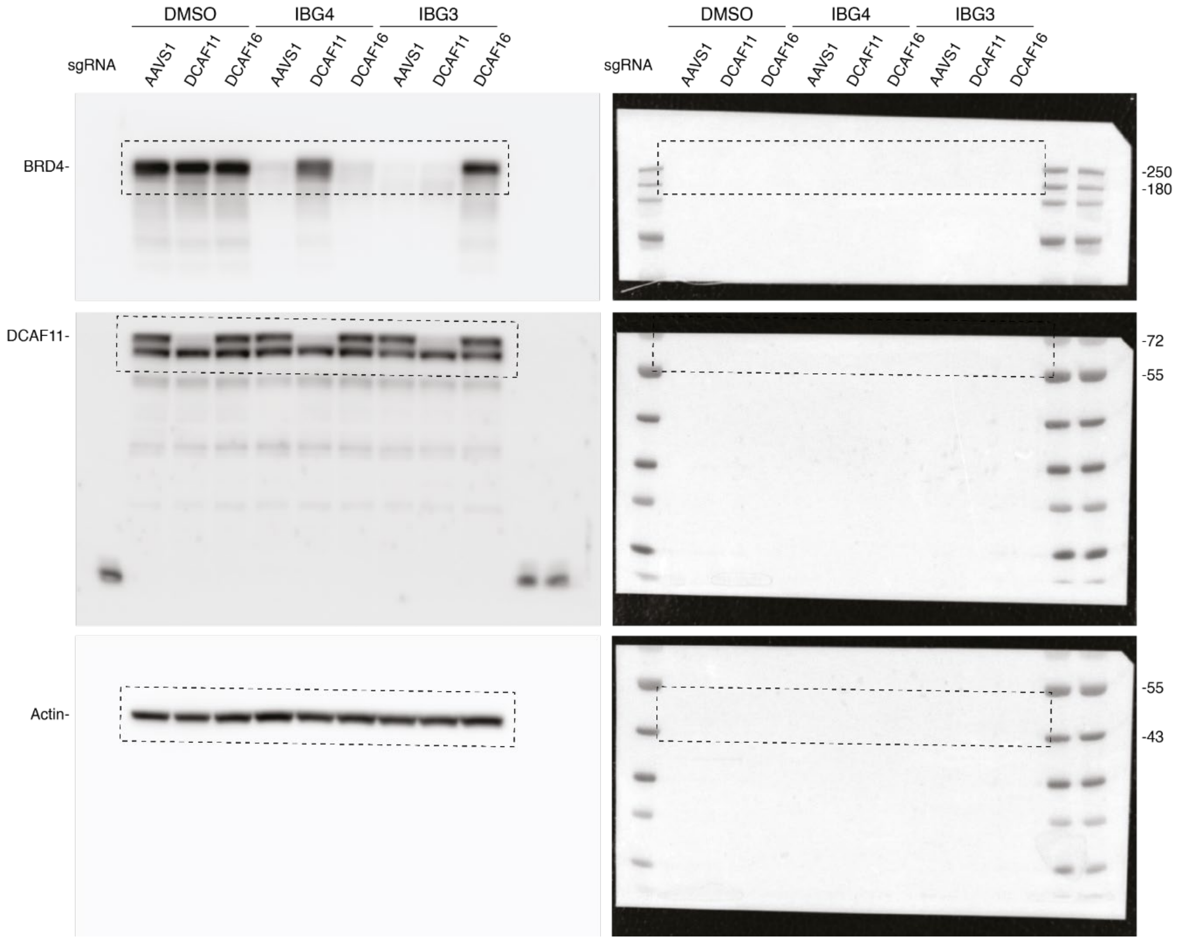

Replicate 2 | Images not shown in the main manuscript.

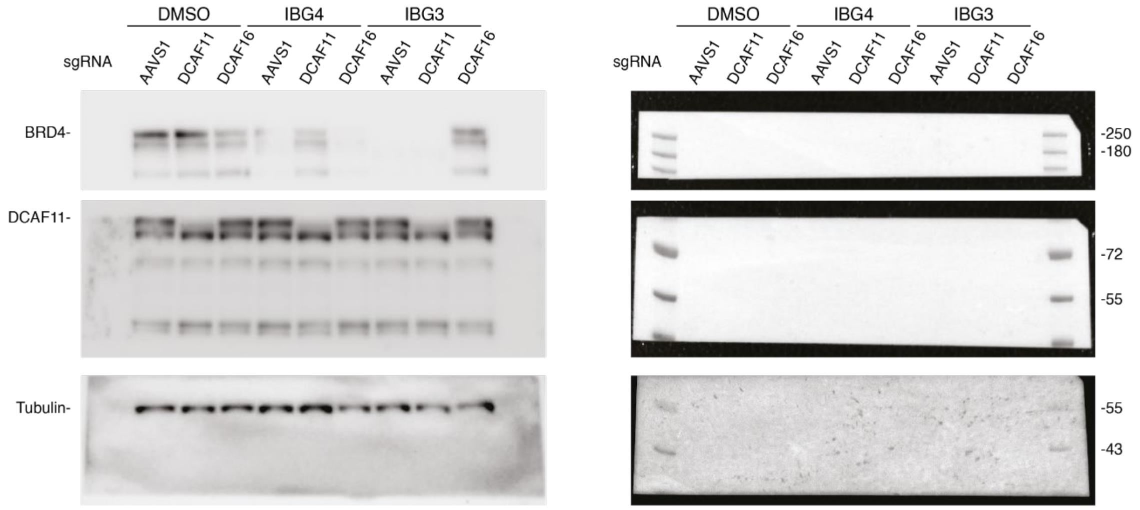

Replicate 3 | Images not shown in the main manuscript.

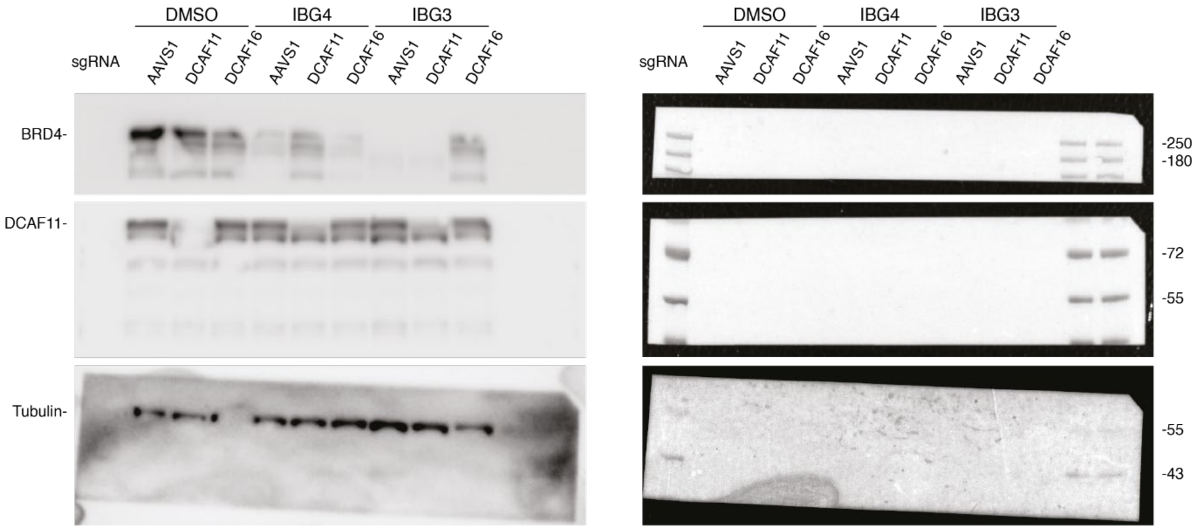

Extended Data Figure 1b.

Boxes indicate regions shown in the figure. Asterisks indicate unspecific bands.

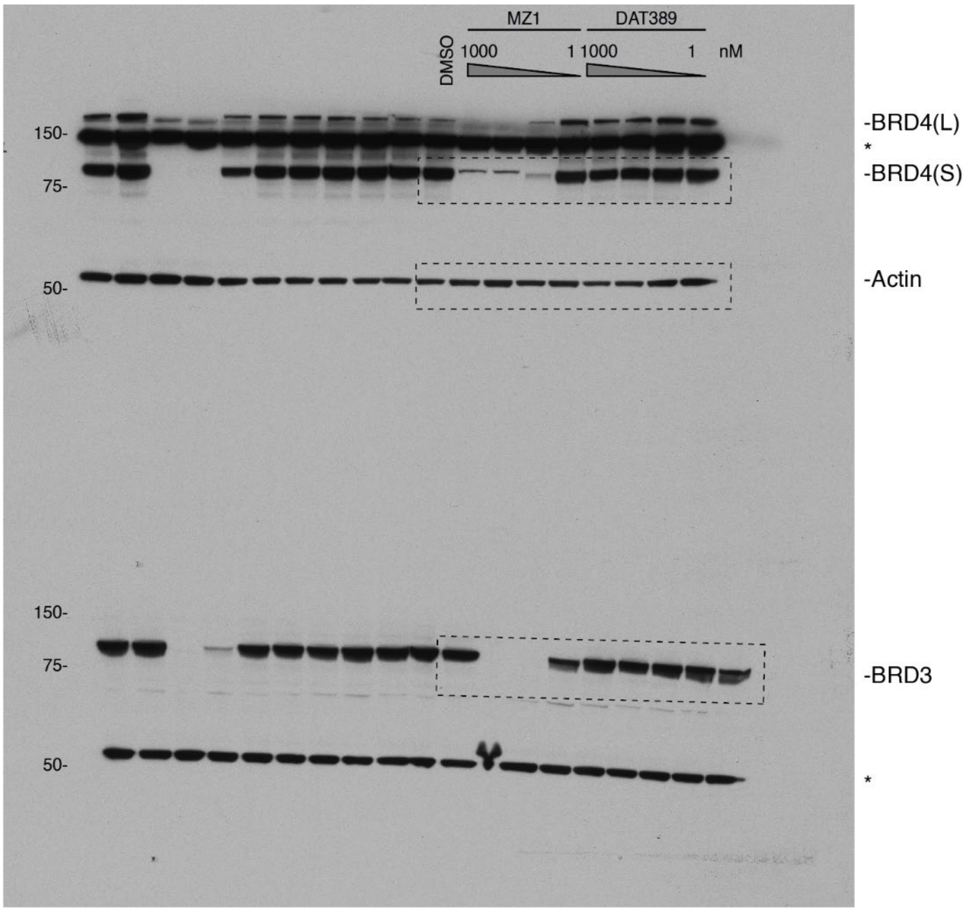

Extended Data Figure 1e.

Replicate 1 | Images were used for quantification but are not shown in the main manuscript. Boxes indicate regions used for quantification. Raw images for Replicate 3 as in Fig. 1b, f.

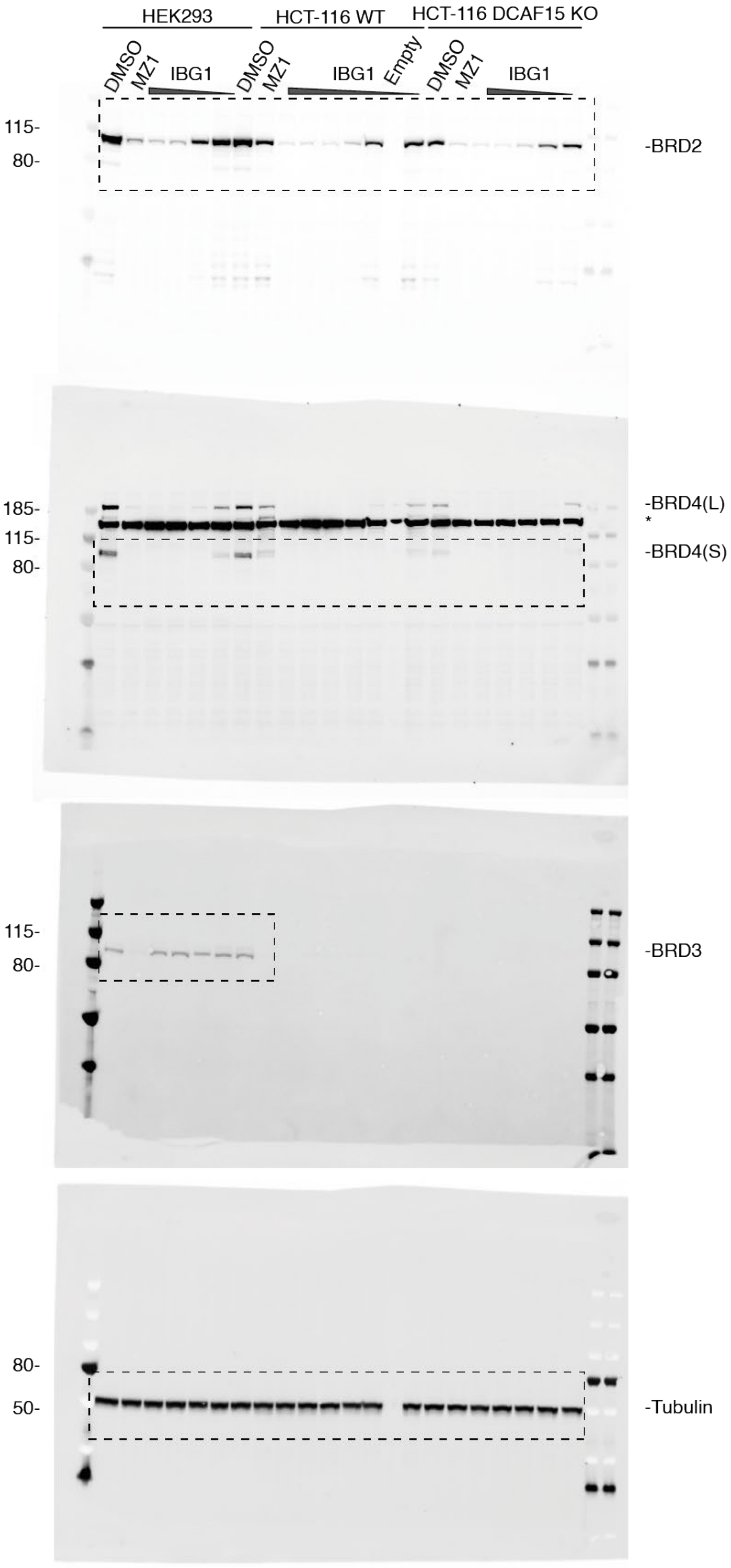

Replicate 2 | Images were used for quantification but are not shown in the main manuscript. Boxes indicate regions used for quantification. Asterisks indicate unspecific bands.

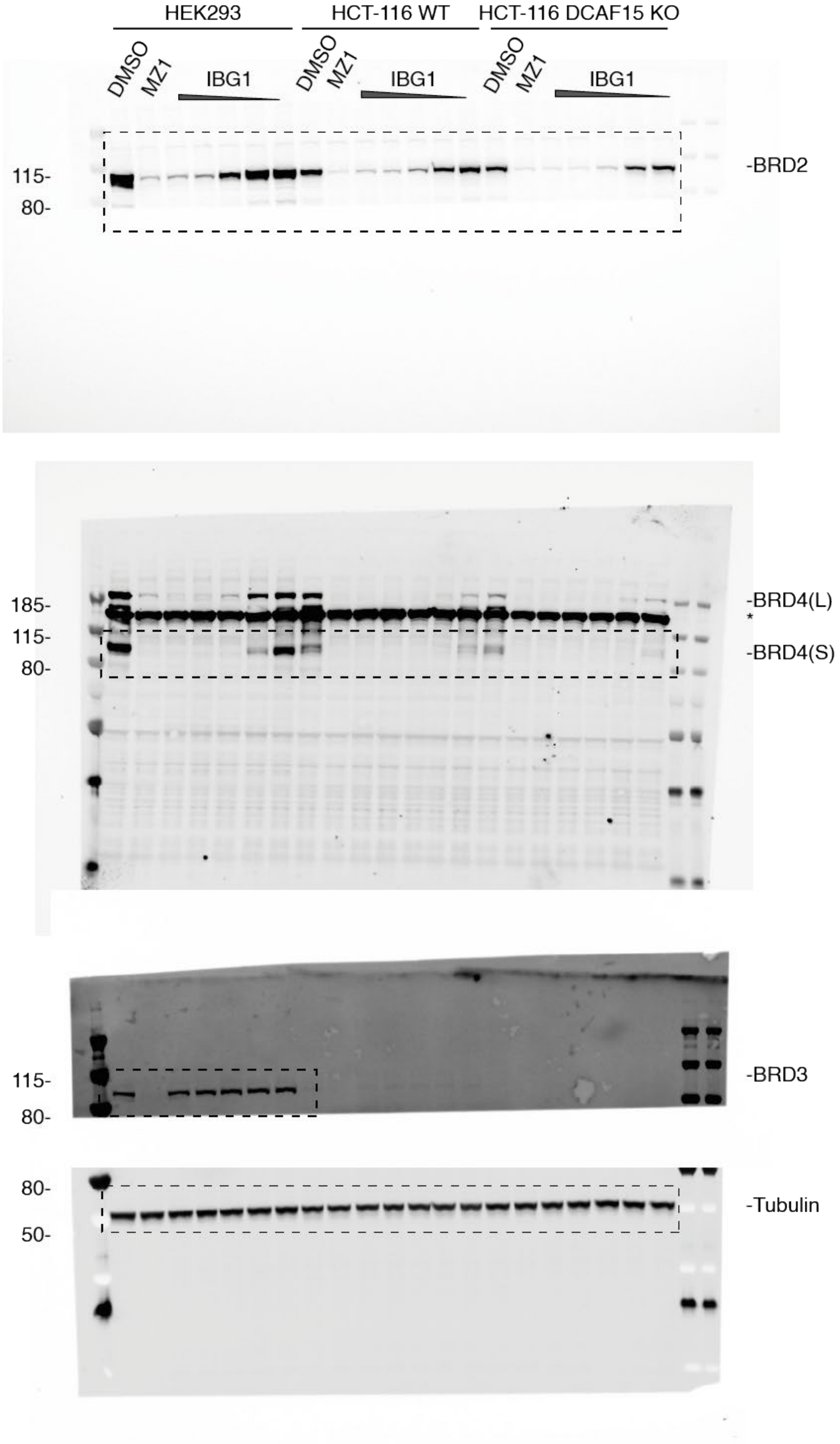

Extended Data Figure 1f.

Boxes inducate regions shown in the figure. Asterisks indicate unspecific bands.

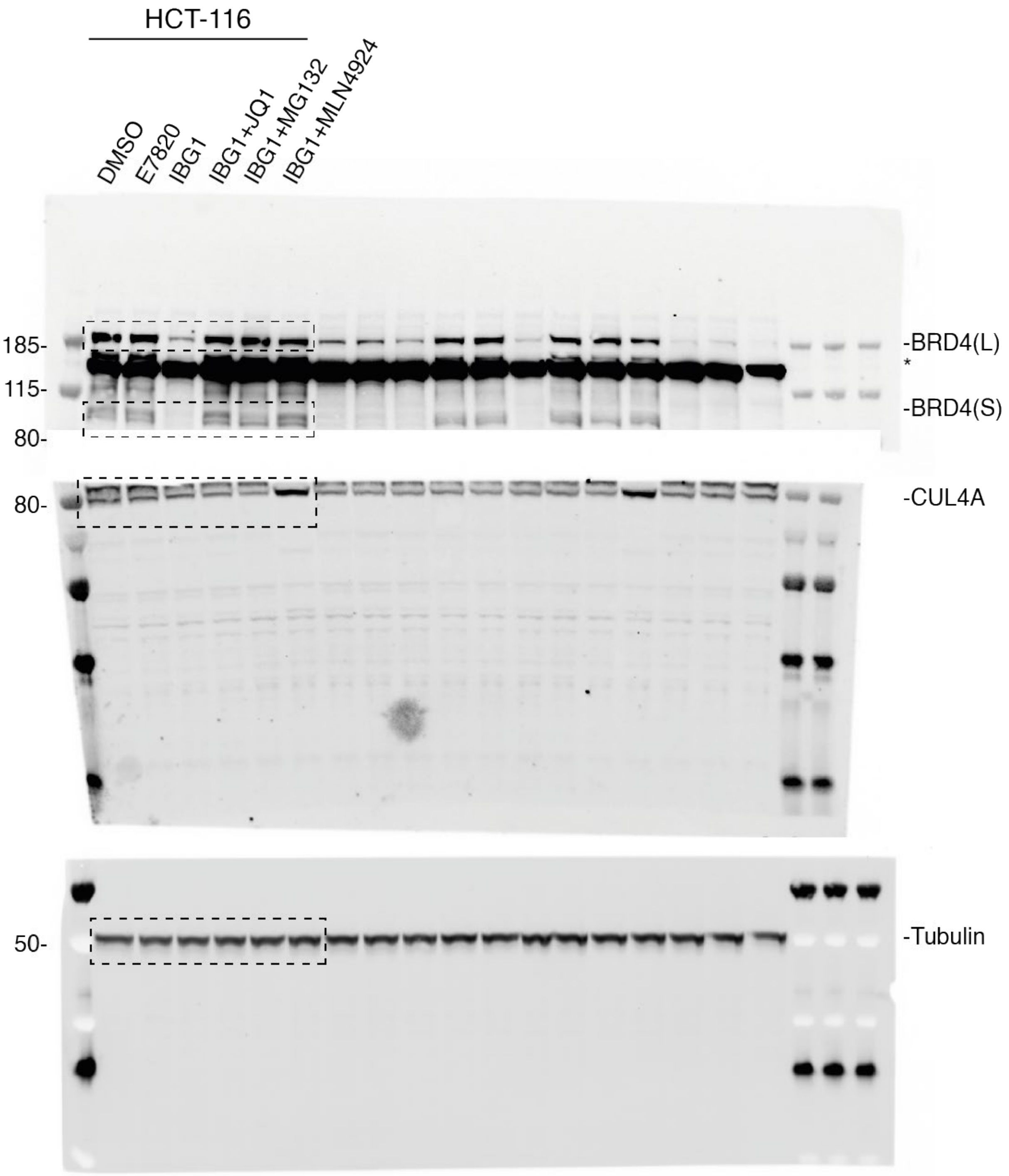

Extended Data Figure 1g.

Boxes inducate regions shown in the figure. Asterisk indicates unspecific band.

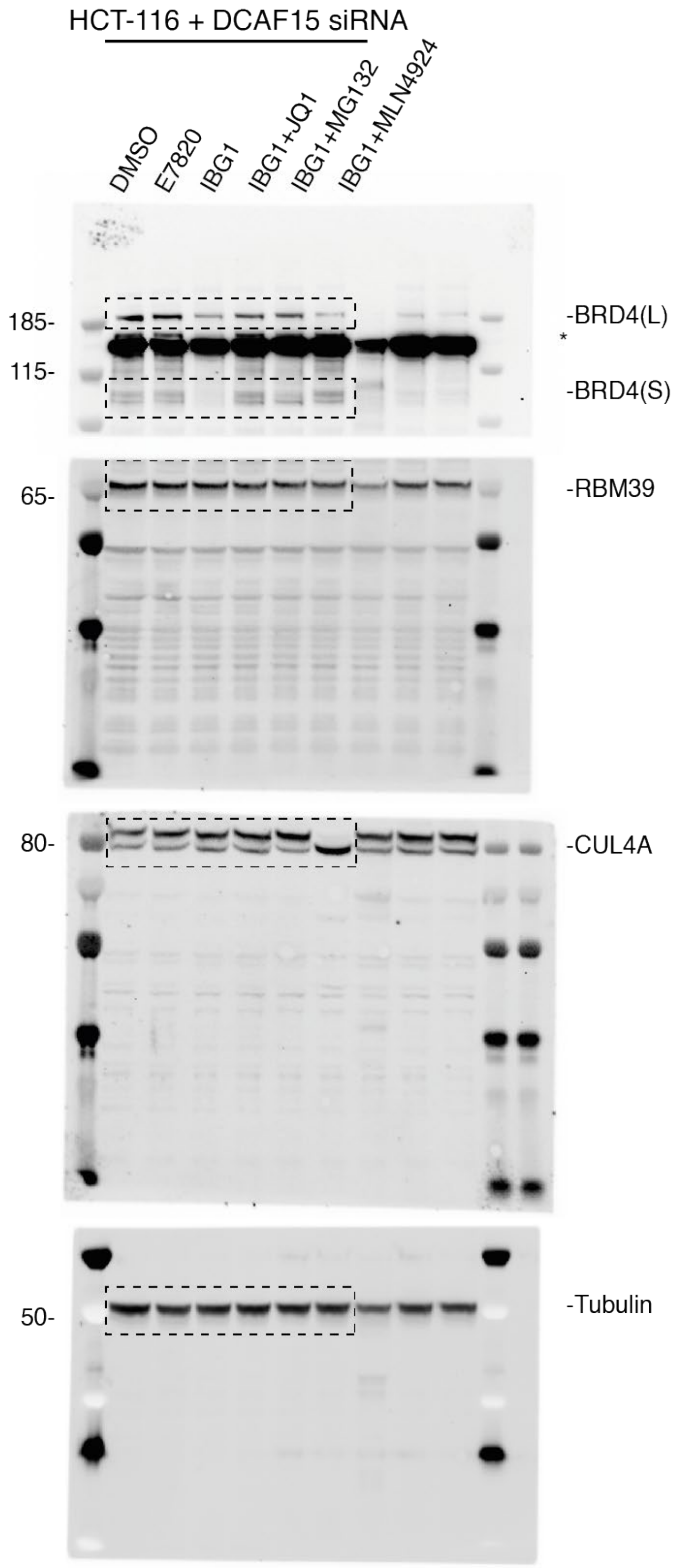

Extended Data Figure 2b.

Replicate 1 | Boxes inducate regions shown in the figure.

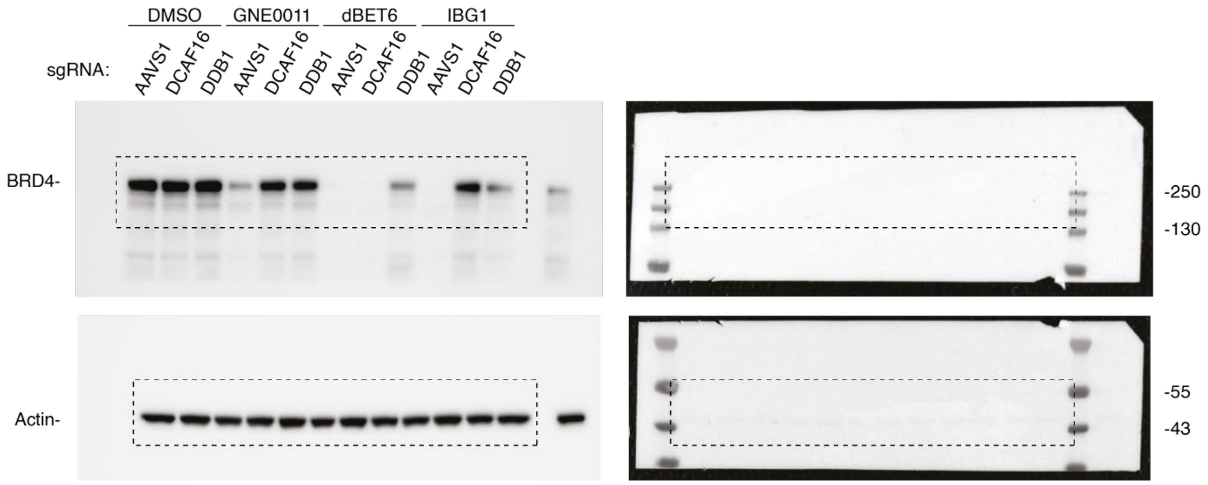

Replicate 2 | Images not shown in the main manuscript.

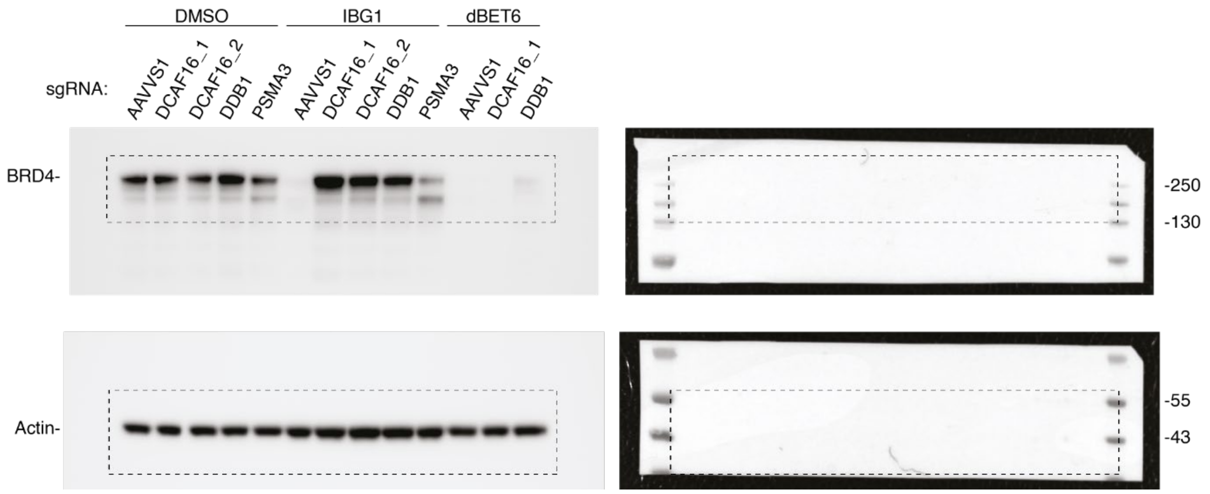

Extended Data Figure 2c.

Boxes inducate regions shown in the figure. Asterisk indicates unspecific band.

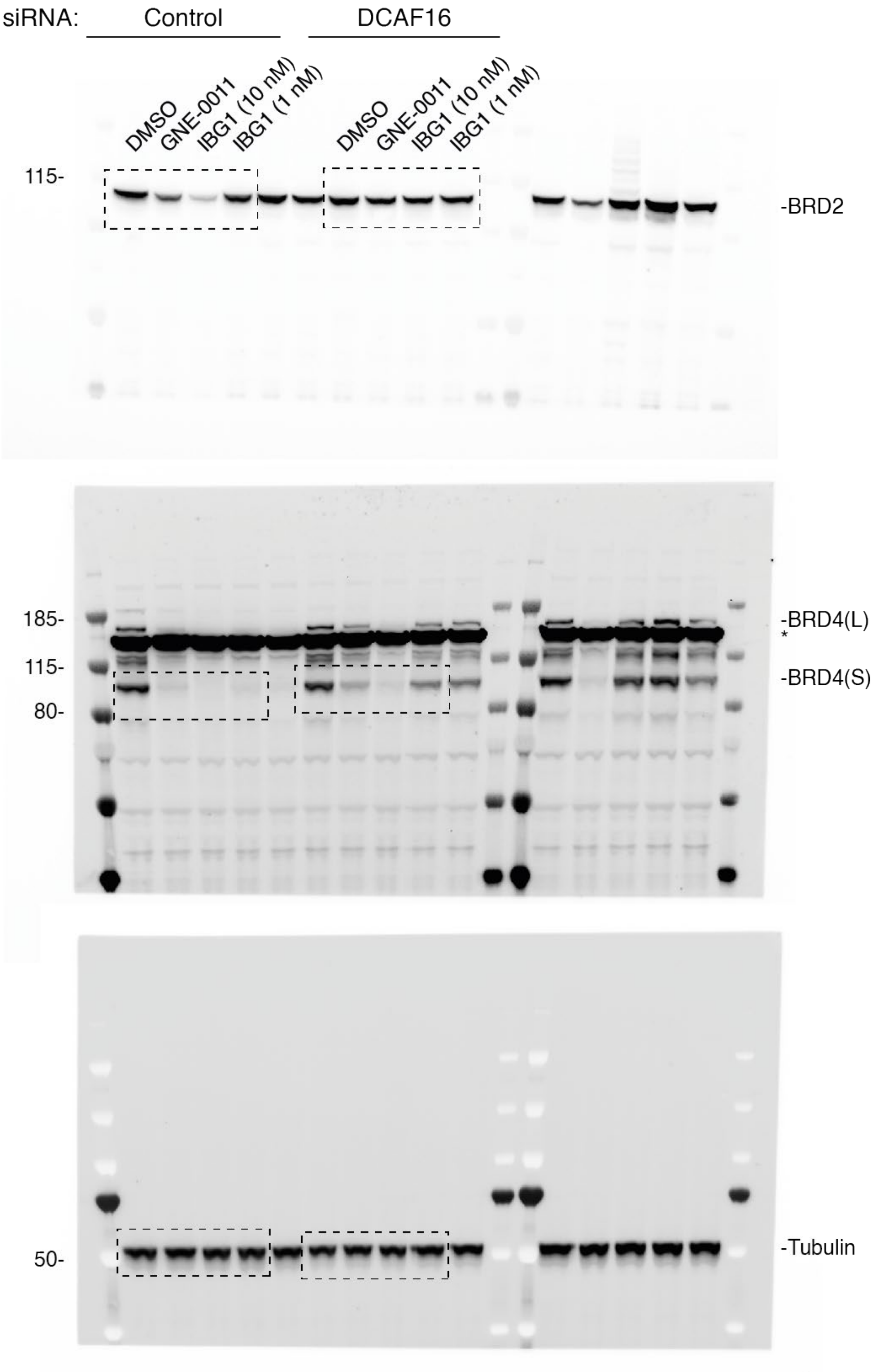

Extended Data Figure 2d.

Boxes inducate regions shown in the figure.

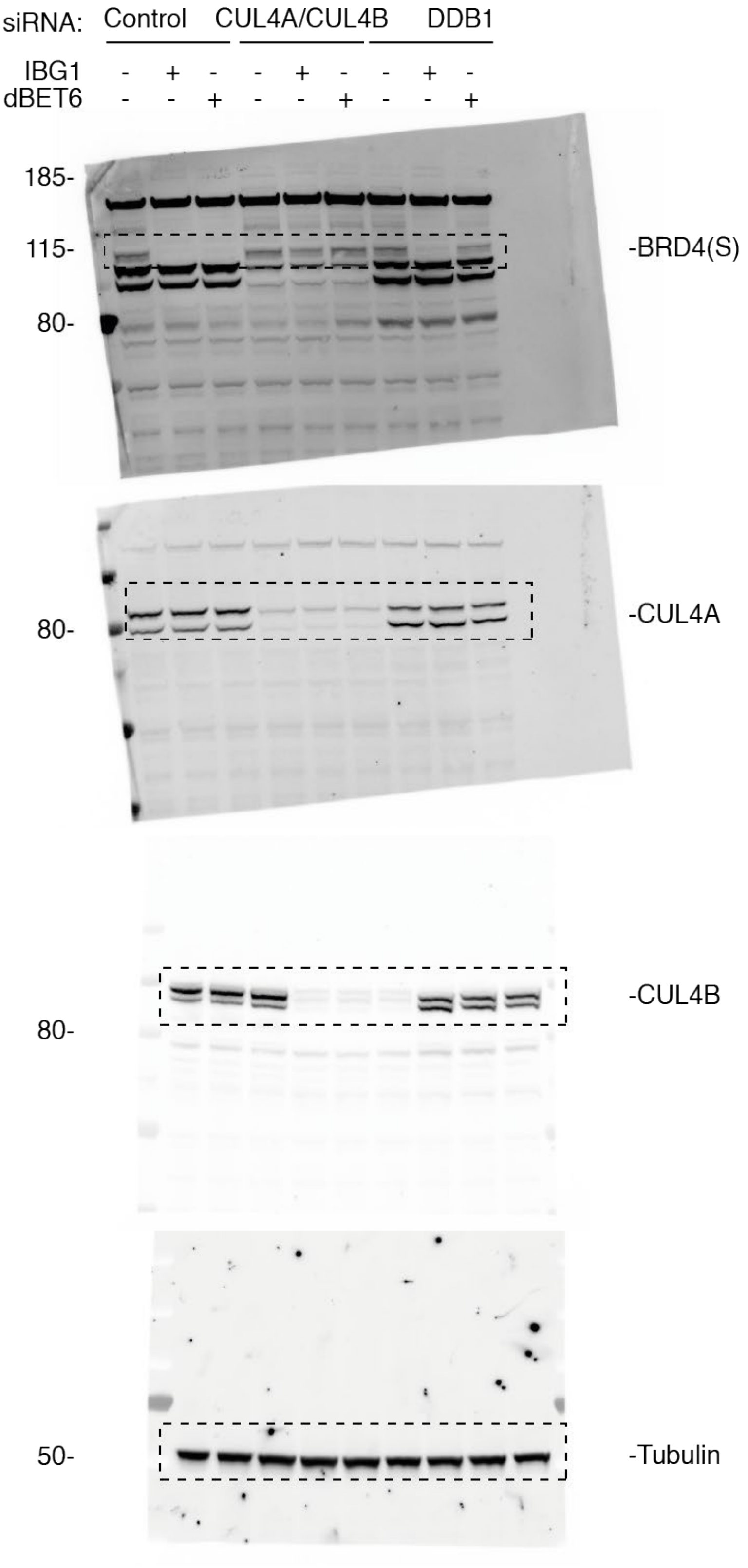

Extended Data Figure 2e.

Boxes inducate regions shown in the figure.

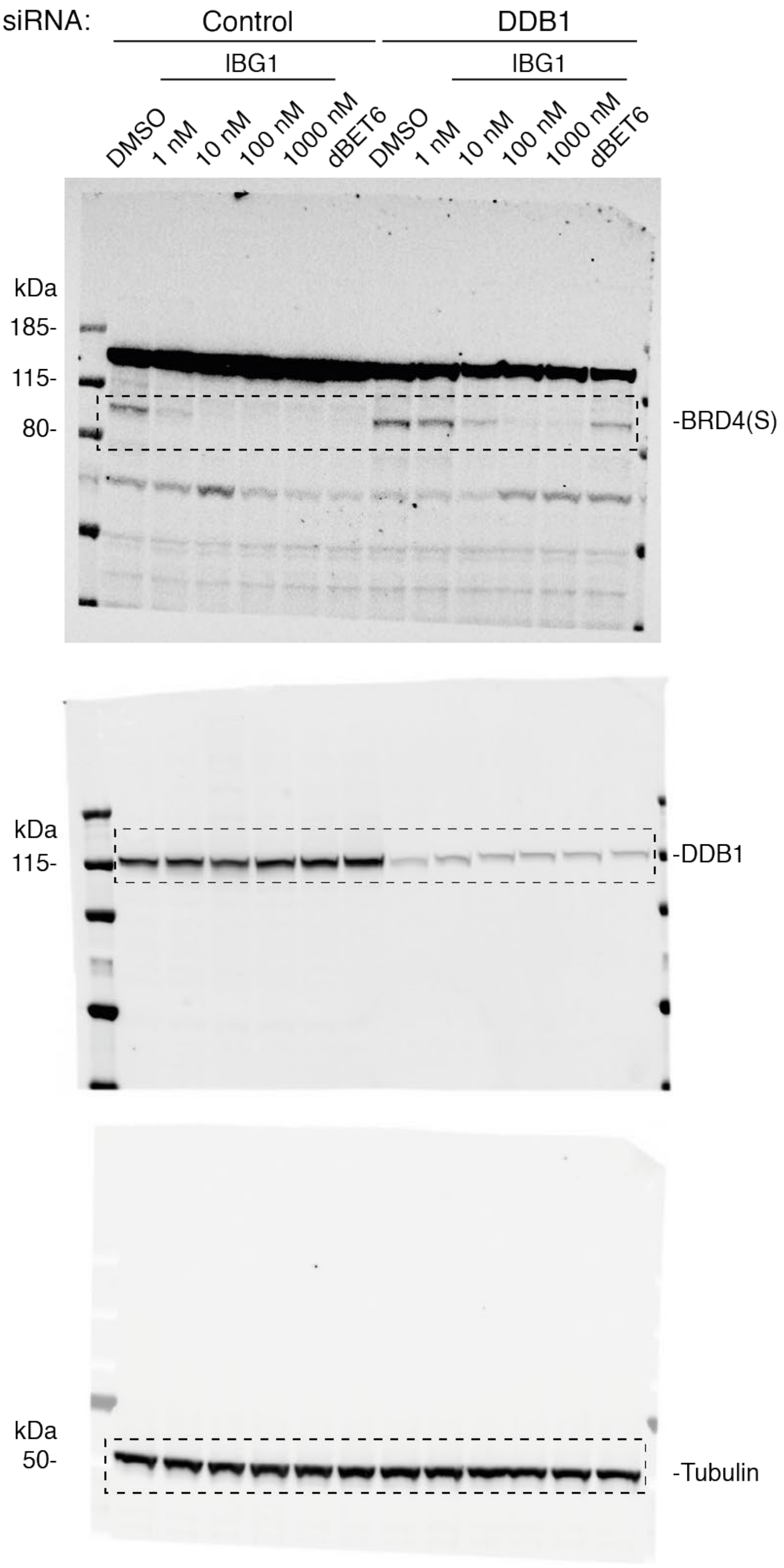

Extended Data Figure 2f.

Boxes inducate regions shown in the figure.

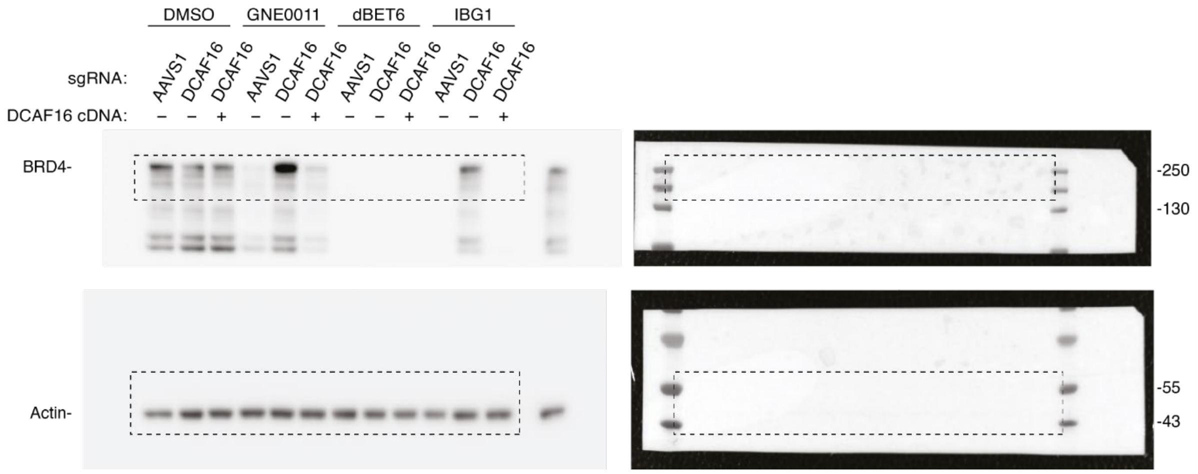

Extended Data Figure 2g.

Replicate 1 | Boxes inducate regions shown in the figure.

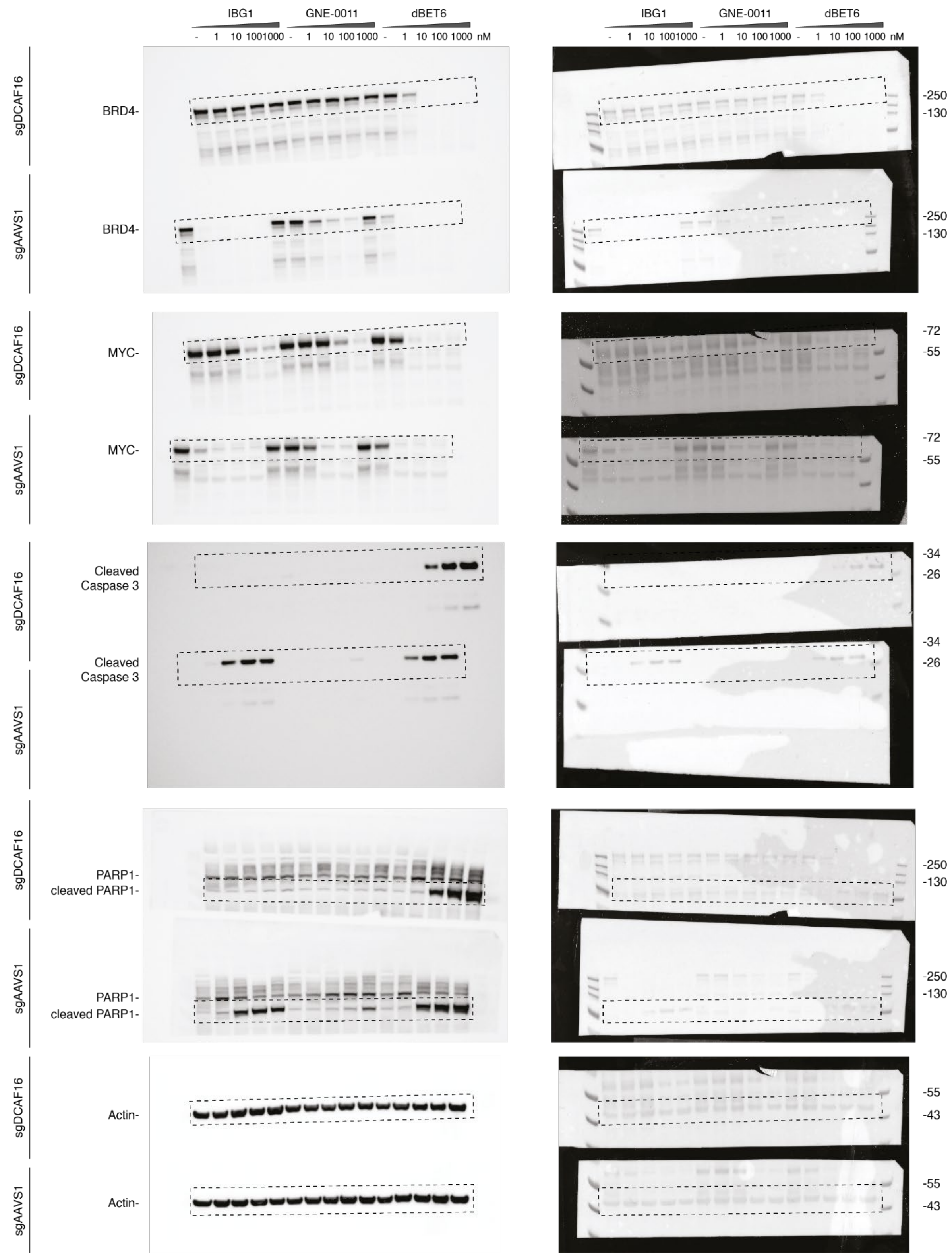

Replicate 2 | Images were used for quantification but are not shown in the main manuscript. Boxes indicate regions used for quantification.

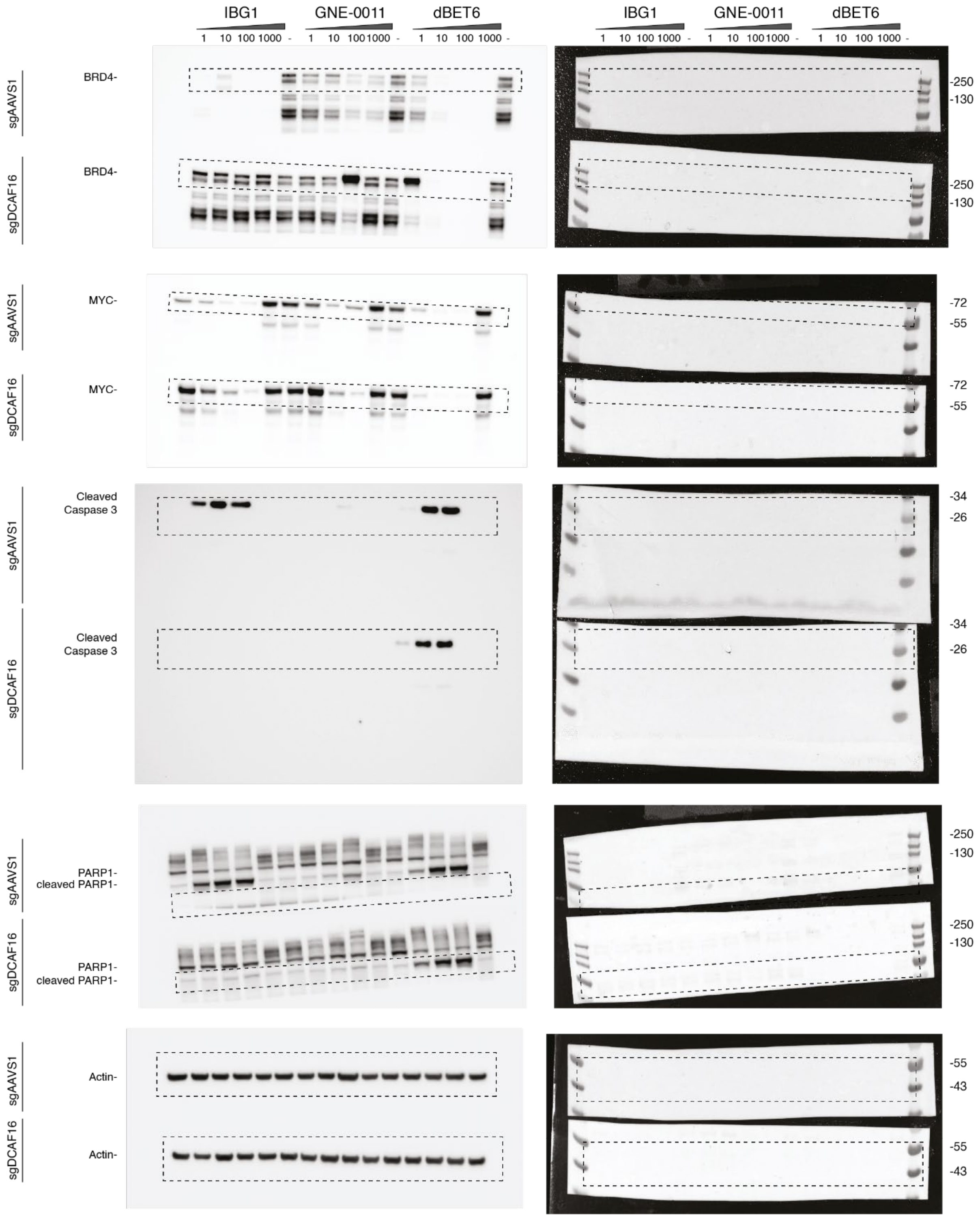

Replicate 3 | Images were used for quantification but are not shown in the main manuscript. Boxes indicate regions used for quantification.

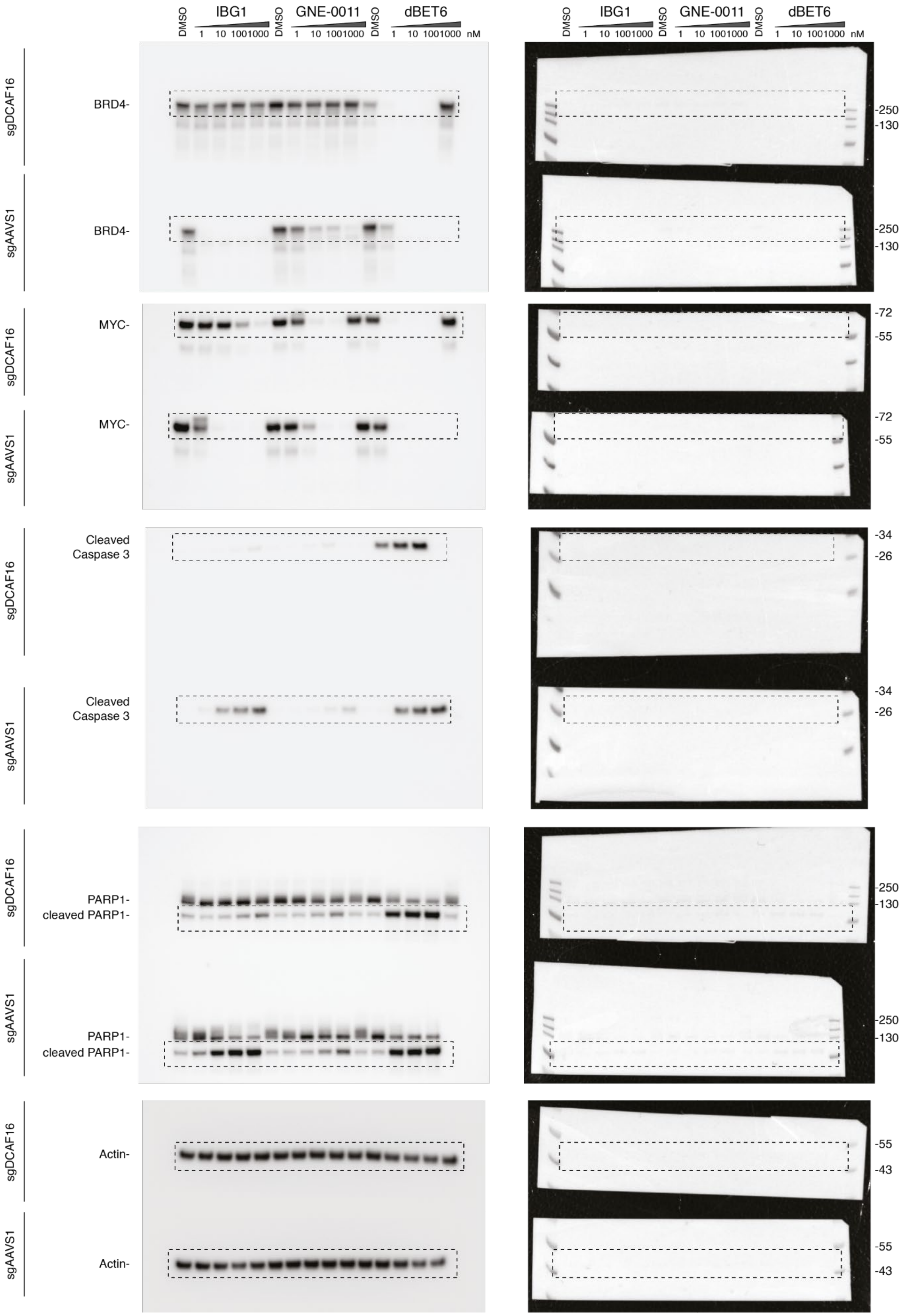

Extended Data Figure 3a.

Boxes inducate regions shown in the figure.

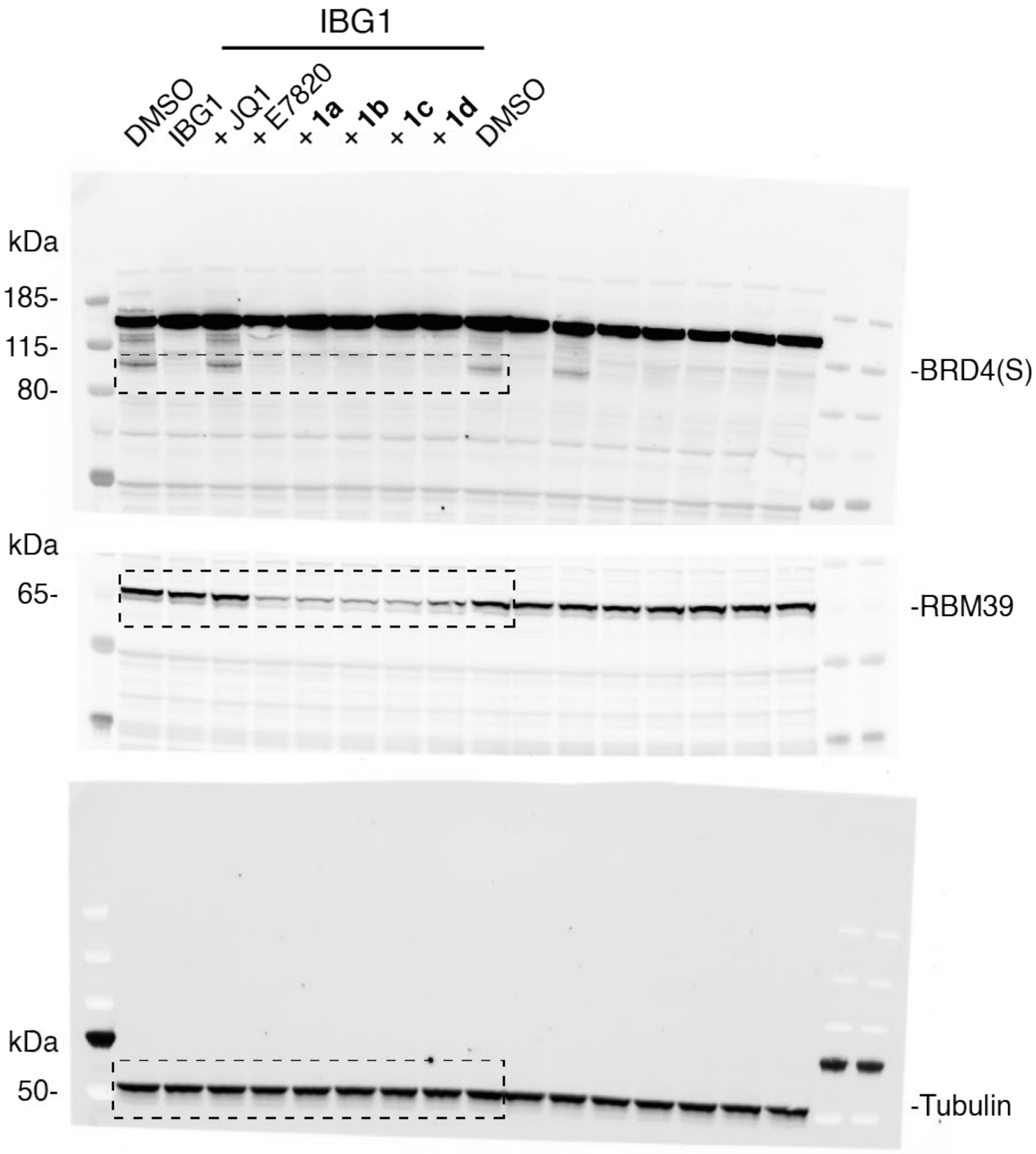

Extended Data Figure 3b.

Boxes inducate regions shown in the figure. Asterisk indicates unspecific band.

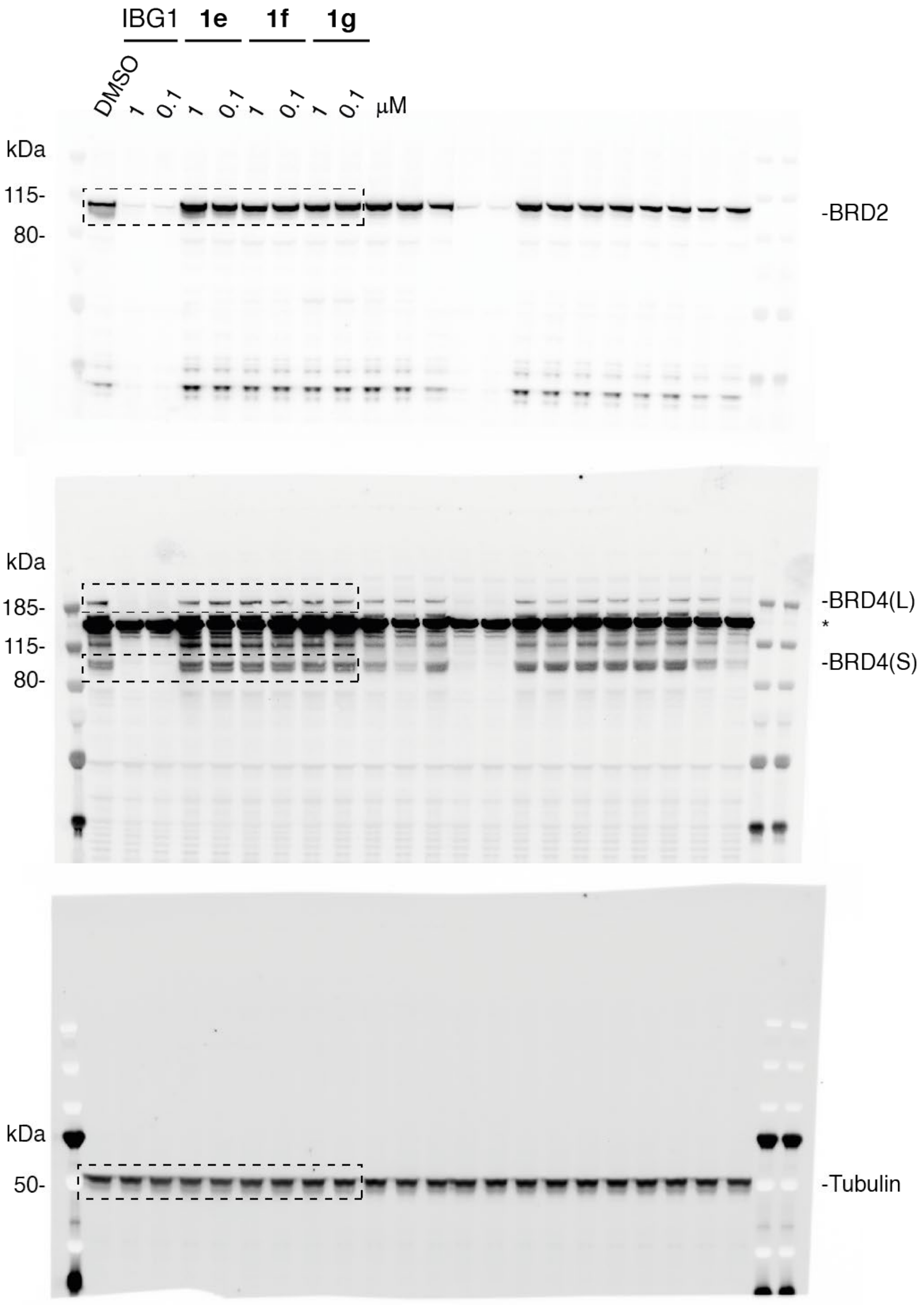

Extended Data Figure 3h.

Boxes inducate regions shown in the figure. Asterisk indicates unspecific band.

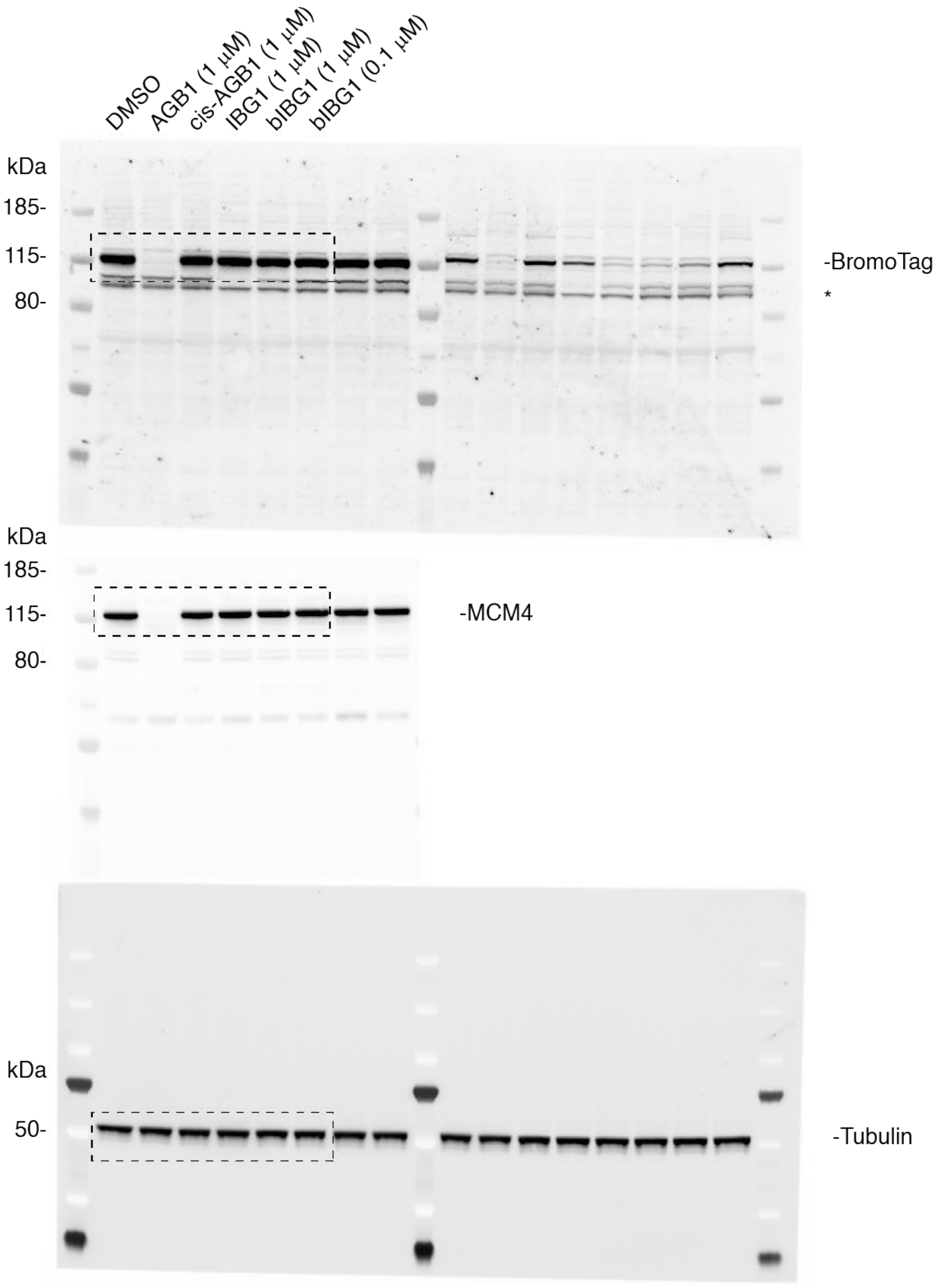

Extended Data Figure 7i.

Boxes inducate regions shown in the figure.

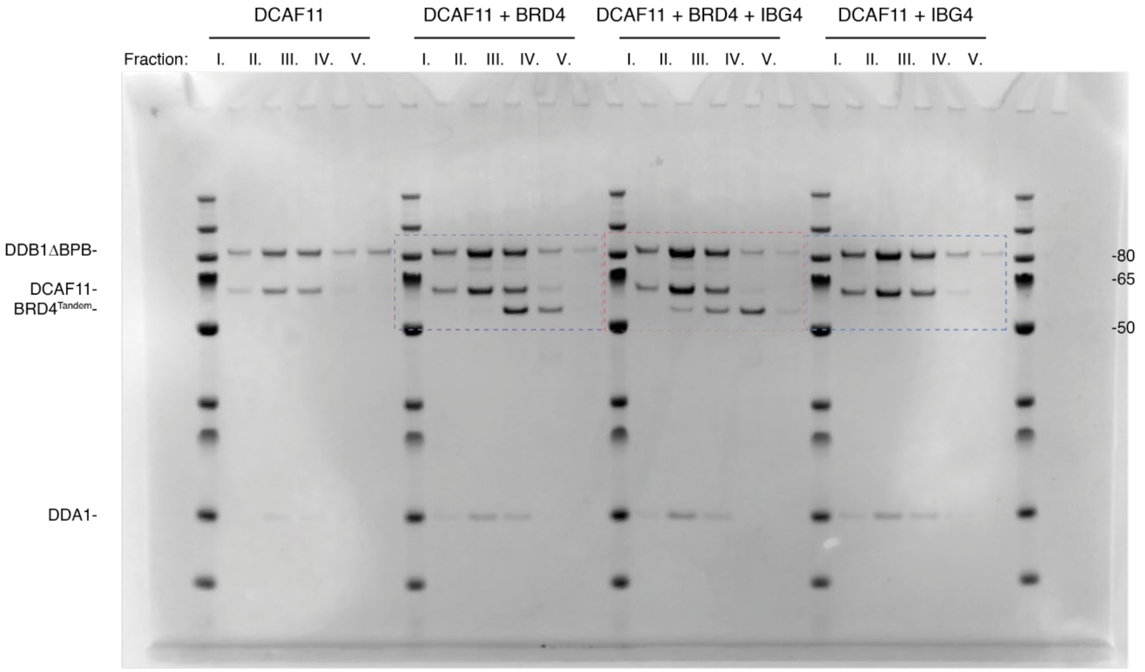

Supplementary Figure 2 | Gating strategies for flow cytometric analyses and cell sorting.

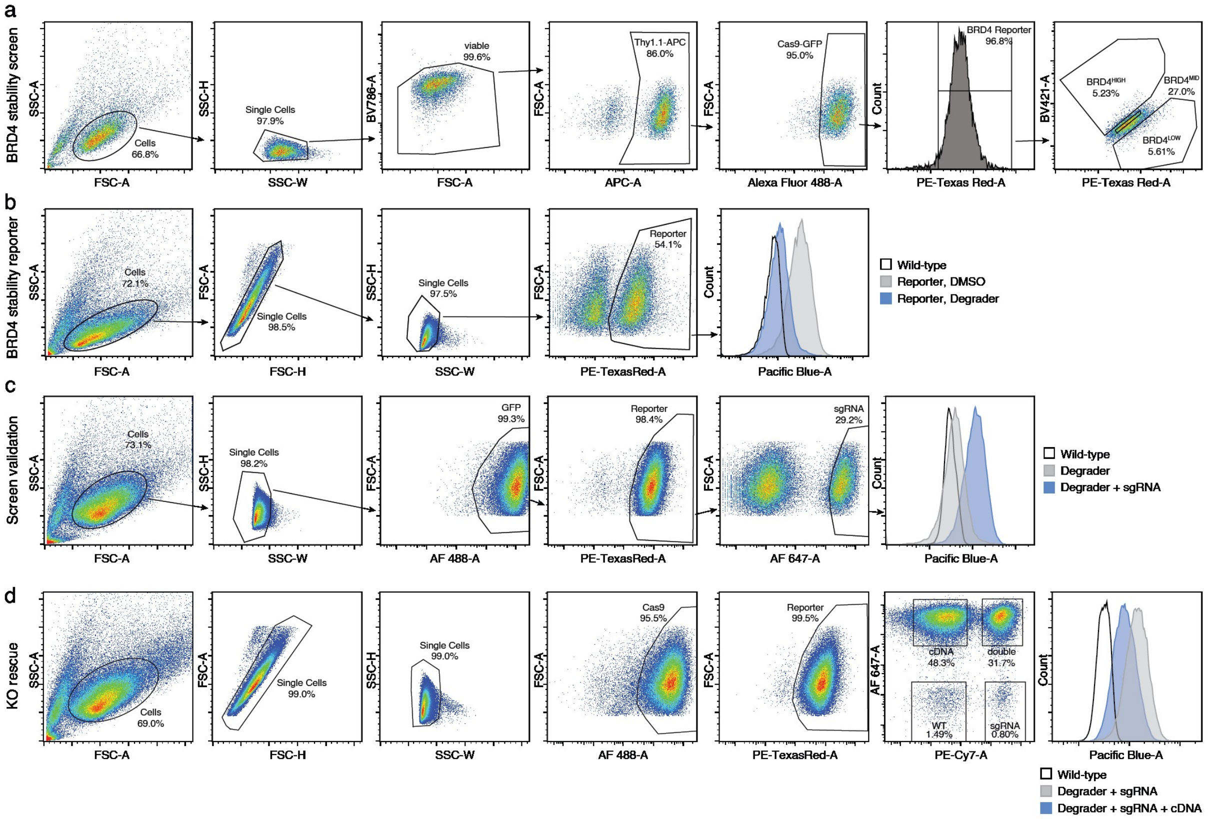

Supplementary Figure 2 | Gating strategies for flow cytometric analyses and cell sorting.

Representative scatter plots of hierarchical gating strategies. **a**, FACS-based BRD4 stability CRISPR screens, as in Fig. 2b, Fig. 5e, Extended Data Fig. 2a, Extended Data Fig. 6f. **b**, BRD4 stability reporter assay, as in Fig. 3f, Fig. 4f, Fig. 4h, Fig. 5b, Extended Data Fig. 3f, Extended Data Fig. 6c, Extended Data Fig. 6e, Extended Data Fig. 6h, Extended Data Fig. 7a, Extended Data Fig. 7d, Extended Data Fig. 7e. **c**, Screen validation BRD4 stability reporter assay, as in Fig. 2d, Extended Data Fig. 6g, Extended Data Fig. 7f. **d**, KO/rescue BRD4 stability reporter assay, as in Fig. 2e. In all analyses, forward scatter area vs. side scatter area plot was used to separate cell events from debris and dead cells. Forward scatter height vs. forward scatter area and/or side scatter width vs. side scatter height plots were used to separate single cells from aggregates. For the sorting of fixed cells in CRISPR BRD4 protein stability screens, dead cells were excluded based on Zombia-NIR staining (BV786-A) vs FSC-A and sgRNA library (Thy1.1-APC-A), iCas9 (FITC-A) and reporter (PE-TexasRed-A) triple positive cells were sorted into $BRD4^{LOW}$, $BRD4^{HIGH}$, and $BRD4^{MID}$ populations based on BRD4-BFP (BV421-A) vs mCherry (PE-TexasRed-A) scatter plots. These gates were dynamically adjusted to keep the percentage at 5-10% for $BRD4^{HIGH}$ and $BRD4^{LOW}$ and 25-30% for $BRD4^{MID}$ populations.

References

10 Zoppi, V. *et al.* Iterative Design and Optimization of Initially Inactive Proteolysis Targeting Chimeras (PROTACs) Identify VZ185 as a Potent, Fast, and Selective von Hippel-Lindau (VHL) Based Dual Degrader Probe of BRD9 and BRD7. *J Med Chem* **62**, 699-726, doi:10.1021/acs.jmedchem.8b01413 (2019).
