# Supplementary material for: Targeted protein degradation via intramolecular bivalent glues
Source: Nature. 2024 Feb 21;627(8002):204–11. doi: 10.1038/s41586-024-07089-6 (PMC10917667; doi:10.1038/s41586-024-07089-6)
Supplement: Supplementary file 2 — Reporting Summary [file 41586_2024_7089_MOESM2_ESM.pdf]

nature portfolio

Corresponding author(s): Alessio Ciulli, Georg Winter

Last updated by author(s): Jan 8, 2024

Reporting Summary

Nature Portfolio wishes to improve the reproducibility of the work that we publish. This form provides structure for consistency and transparency in reporting. For further information on Nature Portfolio policies, see our Editorial Policies and the Editorial Policy Checklist.

Statistics

For all statistical analyses, confirm that the following items are present in the figure legend, table legend, main text, or Methods section.

n/a	Confirmed	
☐	☒	The exact sample size (*n*) for each experimental group/condition, given as a discrete number and unit of measurement
☐	☒	A statement on whether measurements were taken from distinct samples or whether the same sample was measured repeatedly
☐	☒	The statistical test(s) used AND whether they are one- or two-sided<br>*Only common tests should be described solely by name; describe more complex techniques in the Methods section.*
☒	☐	A description of all covariates tested
☐	☒	A description of any assumptions or corrections, such as tests of normality and adjustment for multiple comparisons
☐	☒	A full description of the statistical parameters including central tendency (e.g. means) or other basic estimates (e.g. regression coefficient) AND variation (e.g. standard deviation) or associated estimates of uncertainty (e.g. confidence intervals)
☐	☒	For null hypothesis testing, the test statistic (e.g. *F*, *t*, *r*) with confidence intervals, effect sizes, degrees of freedom and *P* value noted<br>*Give P values as exact values whenever suitable.*
☒	☐	For Bayesian analysis, information on the choice of priors and Markov chain Monte Carlo settings
☒	☐	For hierarchical and complex designs, identification of the appropriate level for tests and full reporting of outcomes
☒	☐	Estimates of effect sizes (e.g. Cohen's *d*, Pearson's *r*), indicating how they were calculated

Our web collection on statistics for biologists contains articles on many of the points above.

Software and code

Policy information about availability of computer code

Data collection	Flow cytometry: Data was collected on a BD LSRFortessa using BD FACSDiva software (v9.0), BD FACSAria Fusion using BD FACSDiva software (v8.0.2)<br>Western blotting/SDS gels: ChemiDoc Touch imaging system (BioRad) operated on Image Lab (v2.4.0.03).<br>NGS: Illumina HiSeq3500 (https://www.illumina.com/)<br>Cell viability assays: PerkinElmer VICTOR X3 operated on PerkinElmer 2030 software (v4.0) or BMG Labtech PHERAstar (firmware v1.33).<br>HiBiT and NanoBRET endpoint assays: BMG Labtech PHERAstar (firmware v1.33)<br>Promega kinetic assays: GloMax® Discover System (software v4.0.0, firmware v4.92)<br>Mass spectrometry: Orbitrap Fusion Lumos Tribrid mass spectrometer coupled to a Dionex Ultimate 3000 RSLCnano system and operated via Xcalibur (v4.3.73.11) and Tune (v3.4.3072.18).<br>Cryo-EM: Glacios Transmission Electron Microscope (Thermo Fisher) with Falcon4i direct electron detector, operated on EPU (v3.0) software.
Data analysis	Flow Cytometry Analysis: Flowjo (v10.8.1)<br>FACS-based CRISPR screens: All relevant software is described in detail in the corresponding methods section. Pipelines for sgRNA quantification and statistical analysis are available on Github (https://github.com/ZuberLab/crispr-processnf/tree/566f6d46bbcc2a3f49f51bbc96b9820f408ec4a3 and https://github.com/ZuberLab/crisprmageck-nf/tree/c75a90f670698bfa78bfd8be786d6e5d6d4fc455). Used packages: fastx-toolkit (v0.0.14), Bowtie2 (v2.4.5), featureCounts (v2.0.1), MAGeCK (v0.5.9).<br>Viability-based CRISPR screen: All relevant software is described in detail in the corresponding methods section. Used packages: bcl2fastq (v2.20.0.422), cutadapt (v2.8), Bowtie2 (v2.3.0), MAGeCK (v0.5.9). SgRNAs for ubiquitin/Nedd8 focused library were selected with the Broad Institute CRISPick tool.<br>Western blot quantification: Image Lab (v6.1 build 7)

Data compiling, processing and statistical analyses: Microsoft Excel for Microsoft 365 (v2208.16.0.15601.20526), R Studio (v2022.12.0 Build 353) with R (v4.2.2), GraphPad Prism (v9.3.1, v9.5.1)
Mass Spectrometry: Proteome Discoverer (v2.4.1.15)
Cryo-EM: Cryosparc (v4.1.2), eLBOW (in Phenix v1.20.1.4487), GRADE web server (Grade2 v1.3.0), ColabFold (v1.3), ModelAngelo (v0.2.2), WinCoot (v0.9.8.1), ISOLDE (v1.6), Phenix (v1.20.1-4487), ChimeraX (v1.6), The PyMOL Molecular Graphics System (v2.5.2)
AlphaFold: model of DCAF11 pulled from https://alphafold.ebi.ac.uk/entry/Q8TEB1 created with AlphaFold Monomer v2.0 pipeline.

Code for analysis of FACS-based screens is available on GitHub (https://github.com/ZuberLab/crispr-process-nf, https://github.com/ZuberLab/crispr-mageck-nf).

For manuscripts utilizing custom algorithms or software that are central to the research but not yet described in published literature, software must be made available to editors and reviewers. We strongly encourage code deposition in a community repository (e.g. GitHub). See the Nature Portfolio guidelines for submitting code & software for further information.

Data

Policy information about availability of data

All manuscripts must include a data availability statement. This statement should provide the following information, where applicable:

- Accession codes, unique identifiers, or web links for publicly available datasets
- A description of any restrictions on data availability
- For clinical datasets or third party data, please ensure that the statement adheres to our policy

Source data for Fig. 1c, Fig. 2b, c, Fig. 5d, Extended Data Fig. 2a and Extended Data Fig. 6f are included in the Supplementary information files of the manuscript (Supplementary Data 1-5). Cryo-EM density maps are deposited in the EMDB with the accession code EMD-17172. The atomic model is deposited under Protein Data Bank ID 8OV6. Quantitative proteomics data have been deposited to the ProteomeXchange Consortium PRIDE repository with the accession ID PXD040570. Crystallographic or electron microscopy structures of substrate receptors bound to DDB1 and of BET inhibitors bound to BRD4 bromodomains shown for comparison in Extended Data Fig. 5b, c were obtained from the RCSB protein database (https://www.rcsb.org/) via accessions 5JK7, 5FQD, 6UD7, 7ZN7, 4E96, 5Y94, 3MXF and 6DUV. Full version of all gels and blots are provided in Supplementary Fig. 1, schematics of gating strategies applied for FACS analyses and cell sorting are provided in Supplementary Fig. 2.
All biological materials are available upon reasonable requests under material transfer agreements (MTA) with The Centre for Targeted Protein Degradation, University of Dundee, or CeMM Research Center for Molecular Medicine of the Austrian Academy of Sciences, respectively.

Human research participants

Policy information about studies involving human research participants and Sex and Gender in Research.

Reporting on sex and gender	N/A
Population characteristics	N/A
Recruitment	N/A
Ethics oversight	N/A

Note that full information on the approval of the study protocol must also be provided in the manuscript.

Field-specific reporting

Please select the one below that is the best fit for your research. If you are not sure, read the appropriate sections before making your selection.

☒ Life sciences ☐ Behavioural & social sciences ☐ Ecological, evolutionary & environmental sciences

For a reference copy of the document with all sections, see nature.com/documents/nr-reporting-summary-flat.pdf

Life sciences study design

All studies must disclose on these points even when the disclosure is negative.

Sample size	Sample sizes were not predetermined using statistical analyses. Sample sizes were based on prior experience in the field and our previous studies (Zengerle et al. ACS Chem Biol 2015; Gadd et al. Nat. Chem. Biol. 2017; Riching et al. ACS Chem Biol. 2018).
Data exclusions	In quantitative proteomics, proteins with less than three unique peptides detected were excluded from downstream analysis. For cryo-EM and data filtering is outlined in Extended Data Fig. 4 and for flow cytometry, gating schematics are shown in Supplementary Fig. 2.
Replication	Unless stated in figure legends or method sections, all experiments were done at least twice and the reproduction were successful. The number of technical or biological replicates and independent biological experiments are specified in the respective figure legends.
Randomization	No randomization was performed, as is standard for genetic, biochemical and structural studies. Internal controls were used for quantitative

Randomization	comparisons.
Blinding	No blinding was performed, as no subjective measurements were done.

Reporting for specific materials, systems and methods

We require information from authors about some types of materials, experimental systems and methods used in many studies. Here, indicate whether each material, system or method listed is relevant to your study. If you are not sure if a list item applies to your research, read the appropriate section before selecting a response.

Materials & experimental systems

n/a	Involved in the study	
☐	☒	Antibodies
☐	☒	Eukaryotic cell lines
☒	☐	Palaeontology and archaeology
☒	☐	Animals and other organisms
☒	☐	Clinical data
☒	☐	Dual use research of concern

Methods

n/a	Involved in the study	
☒	☐	ChIP-seq
☐	☒	Flow cytometry
☒	☐	MRI-based neuroimaging

Antibodies

Antibodies used

The following primary antibodies were used for immunoblotting: BRD2 (1:1000, no. Ab139690, Abcam), BRD3 (1:2000, no. Ab50818, Abcam), BRD4 (1:1000, E2A7X, no. 13440, Cell Signaling Technology and no. Ab128874, Abcam), BromoTag (1:1000, no. NBP3-17999, Novus Biologicals), CUL4A (1:2000, no. A300-738A, Bethyl Laboratories), CUL4B (1:2000, no. 12916-1-AP, Proteintech), DDB1 (1:1000, no. A300-462A, Bethyl Laboratories), MCM4 (1:1000, no. ab4459, Abcam) RBM39 (1:1000, no. HPA001591, Atlas Antibodies), RBX1 (1:1000, D3J5I, no. 11922, Cell Signalling Technology), DCAF11 (1:2000, no. A15519, ABclonal), cleaved Caspase-3 (1:1000, D3E9, no. 9579, Cell Signalling Technology), PARP1 (1:1000, no. 9542, Cell Signalling Technology), MYC (1:500, D84C12, no. 5605, Cell Signalling Technology), β-Actin (1:10000, AC-15, no. A5441, Sigma-Aldrich), α-Tubulin (1:500, DM1A, no. T9026, Sigma-Aldrich).

The following secondary antibodies were used for immunoblotting: HRP anti-rabbit IgG (1:2500, 7074, Cell Signaling Technology), HRP anti-mouse IgG (1:5000, 7076, Cell Signaling Technology), IRDye® 680RD anti-mouse (1:5000, no. 926-68070, Li-Cor), IRDye® 800CW anti-rabbit (1:5000, no. 926-32211, Li-Cor), StarBright™ blue 520 goat anti-mouse (1:5000, no. 12005866, Biorad) and hFABTM rhodamine anti-tubulin (1:5000, no. 12004165, Biorad).

The following antibodies were used for FACS analysis and cell sorting: APC anti-mouse CD90.1/Thy-1.1 antibody (1:400, no. 202526, BioLegend), Human TruStain FcX™ Fc Receptor Blocking Solution (1:400, no. 422302, BioLegend).

Validation

Target specificity for the following antibodies were previously confirmed by the Ciulli group:
- anti-BRD2 (abcam no. ab139690): Knockdown validated, disappearance of the band in immunoblotting upon MZ1 / siRNA treatment (Zengerle et al. ACS Chem. Biol. 2015).
- anti-BRD3 (abcam no. ab50818): Knockdown validated, disappearance of the band in immunoblotting upon MZ1 / siRNA treatment (Zengerle et al. ACS Chem. Biol. 2015).
- anti-BRD4 (abcam no. ab128874): Knockdown validated, disappearance of the bands in immunoblotting upon MZ1 / siRNA treatment (Zengerle et al. ACS Chem. Biol. 2015)

Target specificity for the following antibodies were confirmed with established degrader compounds:
BromoTag (no. NBP3-17999, Novus Biologicals; AGB1 - Bond et al. JMedChem 2021), RBM39 (no. HPA001591, Atlas Antibodies; E7820; Uehara et al. NCB, 2017), BRD4 (2A7X, no. 13440, Cell Signaling Technology; dBET6 - Winter et al. Mol Cell, 2017), MCM4 (no. ab4459, Abcam; BromoTag degrader ABG1).

Target specificity for CUL4A (no. A300-738A, Bethyl Laboratories), CUL4B (no. 12916-1-AP, Proteintech), DDB1 (no. A300-462A, Bethyl Laboratories) and RBX1 antibodies (D3J5I, no. #11922, Cell Signalling Technology) were confirmed by western blotting following corresponding siRNA treatment (48h).

Target specificity for MYC antibody (D84C12, no. 5605, Cell Signalling Technology) was confirmed in previous work by CRISPR-based knockout (De Almeida et al. Nature, 2021).

Target specificity for DCAF11 antibody (no. A15519, ABclonal) was confirmed by western blot following inducible knockout in KBM7 iCas9 cells (Fig. 5e).

Target specificity for Cleaved Caspase 3 (D3E9, no. 9579, Cell Signalling Technology) and PARP1 antibodies was validated by the vendor via control compounds (Etoposide, Stautosporine) and blocking peptides.

Target specificity for APC anti-mouse CD90.1/Thy-1.1 antibody (no. 202526, BioLegend) was verified by ectopic overexpression.

Eukaryotic cell lines

Policy information about cell lines and Sex and Gender in Research

Cell line source(s)	Human HEK293, HCT-116, MV-4-11 and HeLa cells were obtained from ATCC.<br>KBM7 (originally obtained from Haplogen Bioscience) iCas9 cells were a gift from Johannes Zuber (IMP - Research Institute of Molecular Pathology).<br>Lenti-X 293T cells were purchased from Clontech.
Authentication	All used cell lines were authenticated by short tandem repeat (STR) profiling. Successfull CRISPR-based editing of cell lines was confirmed by cell-based degradation assays (RBM39 treatment for the DCAF15 KO cell line or dBET6 treatment for HiBiT-BET cell lines) as well as by Sanger Sequencing.
Mycoplasma contamination	All used cell lines were routinely tested and confirmed negative for mycoplasma contamination.
Commonly misidentified lines (See ICLAC register)	No commonly misidentified cell lines were used.

Flow Cytometry

Plots

Confirm that:

☒ The axis labels state the marker and fluorochrome used (e.g. CD4-FITC).

☒ The axis scales are clearly visible. Include numbers along axes only for bottom left plot of group (a 'group' is an analysis of identical markers).

☒ All plots are contour plots with outliers or pseudocolor plots.

☒ A numerical value for number of cells or percentage (with statistics) is provided.

Methodology

Sample preparation

Sample preparation for each experiment is described in detail in the methods section.

To engineer a BRD4 protein stability reporer, KBM7 iCas9 cells were transduced with lentivirus expressing SFFV-BRD4(S)-mTagBFP-P2A-mCherry to generate stable reporter cell lines. BRD4/mCherry double positive cells were sorted on a Cytoflex SRT (Beckman Coulter). For evaluation of BRD4-TagBFP reporter degradation, cells were treated with DMSO or BRD4 degraders for 6 hours before flow cytometry analysis on an LSRFortessa (BD Biosciences).

For BRD4 stability CRISPR screens library transduced cells were induced with doxycycline for 3 days and harvested after 6 hours of treatment with DMSO or degraders. Cells were washed with PBS, stained with Zombie NIR™ Fixable Viability Dye (1:1000, BioLegend) and APC anti-mouse CD90.1/Thy-1.1 antibody (1:400, BioLegend) in the presence of Human TruStain FcX™ Fc Receptor Blocking Solution (1:400, BioLegend), and fixed with 0.5 mL methanol-free paraformaldehyde 4% (Thermo Scientific™ Pierce™) for 30 min at 4 ºC, while protected from light. Cells were washed with, and stored in FACS buffer (PBS containing 5% FBS and 1 mM EDTA) at 4 ºC over night. The next day, cells were strained trough a 35 µm nylon mesh and sorted on a BD FACSAria™ Fusion (BD Biosciences) using a 100 µm nozzle. Aggregates, dead (ZombieNIR positive), Cas9-negative (GFP) and sgRNA library-negative (Thy1.1-APC) cells were excluded, and the remaining cells were sorted based on their BRD4-BFP and mCherry levels into BRD4HIGH (8-10% of cells), BRD4MID (25-30%) and BRD4LOW (8-10%) fractions. For each sample, cells corresponding to at least 1,500-fold library representation were sorted per replicate.

To validate screen hits, BRD4 reporter cells were lentivirally transduced with an sgRNA expression plasmid (pLenti-U6-sgRNA-IT-EF1αs-Thy1.1-P2A-NeoR) at 30-50% transduction efficiency. Cas9 expression was induced with docycycline (0.4 µg ml−1) for 3days, followed by 6 hours of degrader treatment. Cells were stained for sgRNA expression with an APC conjugated anti-mouse -CD90.1/Thy1.1 antibody (#202526, Biolegend; 1:400) and Human TruStain FcX Fc receptor blocking solution (#422302, Biolegend; 1:400) for 5 minutes in FACS buffer (PBS containing 5% FBS and 1 mM EDTA) at 4°C. Cells were washed and resuspended in FACS buffer and analyzed on an LSRFortessa (BD Biosciences).

For KO/rescue studies, sgDCAF16 expressing BRD4-reporter KBM7 iCas9 cells were transduced with a lentivirus expressing an sgRNA resistant DCAF16 cDNA (pRRL-SFFV-3xFLAG-DCAF16-EF1αs-iRFP670) and analyzed as above.
For BRD4 degradation studies, KBM7 iCas9 cells were lentivirally transduced with mutant or truncated versions of the BRD4-TagBFP protein stability reporter and treated with DMSO or degraders for 6 hours. Reporter negative cells were excluded based on mCherry signal and reporter positive cells were analyzed.
Flow cytometric data analysis was performed in FlowJo v10.8.1. BFP and mCherry mean fluorescence intensity (MFI) values for were normalized by background subtraction of the respective values from reporter-negative KBM7 wild type cells. BRD4 abundance was calculated as the ratio of background subtracted BFP to mCherry MFI, and is displayed normalized to DMSO treated, sgRNA/cDNA double negative cells.

Instrument	All flow cytometric analyses were performed on BD LSRFortessa (4 laser, 16 detector configuration; BD Bioscience). All sorts were performed on BD FACSAria Fusion (5 lasers, 16 detectors; BD Bioscience) or CytoFLEX SRT (4 lasers, 15 detectors; Beckman Coulter) cell sorters.
Software	BD FACSDiva software (v8.0.2 and v9.0), Beckman Coulter CytExpert SRT (v 1.1.0.10007), Flowjo (v10.8.1)

Cell population abundance

In BRD4 stability CRISPR screens, cells were sorted into BRD4HIGH (5-10% of cells), BRD4LOW (5-10%), and BRD4MID (25-30%) populations. All collected fractions were reanalyzed for purity and fractions with > 5% cross-contamination were discarded before further processing.

Gating strategy

In all analyses, forward scatter area vs. side scatter area plot was used to separate cell events from debris and dead cells. Forward scatter height vs. forward scatter area and/or side scatter width vs. side scatter height plots were used to separate single cells from aggregates.
For the sorting of fixed cells in CRISPR BRD4 protein stability screens, dead cells were excluded based on Zombia-NIR staining (BV786-A) vs FSC-A and sgRNA library (Thy1.1-APC-A), iCas9 (FITC-A) and reporter (PE-TexasRed-A) triple positive cells were sorted into BRD4LOW, BRD4HIGH, and BRD4MID populations based on BRD4-BFP (BV421-A) vs mCherry (PE-TexasRed-A) scatter plots. These gates were dynamically adjusted to keep the percentage at 5-10% for BRD4HIGH and BRD4LOW and 25-30% for BRD4MID populations.
A figure exemplifying the gating strategy for all FACS experiments and FACS-based screens in provided in Supplementary Figure 2.

☒ Tick this box to confirm that a figure exemplifying the gating strategy is provided in the Supplementary Information.
